# Figure-ground responsive fields of monkey V4 neurons estimated from natural image patches

**DOI:** 10.1371/journal.pone.0268650

**Published:** 2022-06-16

**Authors:** Kouji Kimura, Atsushi Kodama, Yukako Yamane, Ko Sakai

**Affiliations:** 1 Department of Computer Science, University of Tsukuba, Tsukuba, Japan; 2 Neural Computation Unit, Okinawa Institute of Science and Technology, Okinawa, Japan; University of Minnesota, UNITED STATES

## Abstract

Neurons in visual area V4 modulate their responses depending on the figure-ground (FG) organization in natural images containing a variety of shapes and textures. To clarify whether the responses depend on the extents of the figure and ground regions in and around the classical receptive fields (CRFs) of the neurons, we estimated the spatial extent of local figure and ground regions that evoked FG-dependent responses (RF-FGs) in natural images and their variants. Specifically, we applied the framework of spike triggered averaging (STA) to the combinations of neural responses and human-marked segmentation images (FG labels) that represent the extents of the figure and ground regions in the corresponding natural image stimuli. FG labels were weighted by the spike counts in response to the corresponding stimuli and averaged over. The bias due to the nonuniformity of FG labels was compensated by subtracting the ensemble average of FG labels from the weighted average. Approximately 50% of the neurons showed effective RF-FGs, and a large number exhibited structures that were similar to those observed in virtual neurons with ideal FG-dependent responses. The structures of the RF-FGs exhibited a subregion responsive to a preferred side (figure or ground) around the CRF center and a subregion responsive to a non-preferred side in the surroundings. The extents of the subregions responsive to figure were smaller than those responsive to ground in agreement with the Gestalt rule. We also estimated RF-FG by an adaptive filtering (AF) method, which does not require spherical symmetry (whiteness) in stimuli. RF-FGs estimated by AF and STA exhibited similar structures, supporting the veridicality of the proposed STA. To estimate the contribution of nonlinear processing in addition to linear processing, we estimated nonlinear RF-FGs based on the framework of spike triggered covariance (STC). The analyses of the models based on STA and STC did not show inconsiderable contribution of nonlinearity, suggesting spatial variance of FG regions. The results lead to an understanding of the neural responses that underlie the segregation of figures and the construction of surfaces in intermediate-level visual areas.

## Introduction

Segregation of a natural image into objects and background is a fundamental function in scene understanding. A pixelwise retinal image is segmented and grouped to construct objects in the cortex. Intermediate-level visual cortices play a crucial role in generating figure-ground (FG)-dependent signals. Neurons in V2 have been reported to exhibit selectivity for border ownership, which provides the direction of figure along a contour [1]. Recent studies on texture segregation have suggested distinct processes for the enhancement of figures and suppression of background in early- to intermediate-level visual cortices such as V1 and V4 [2, 3]. In the detection of contours, V4 neurons were reported to exhibit enhanced and suppressed responses to line segments that form global contour and background line segments, respectively [4]. Investigations on curvature coding in V4 have reported that neurons were selectively responded to the preferred curvature along a closed shape and a figural surface [5–7]. A long history of studies has highlighted the variety and complexity of V4 responses including those modulated by color [8, 9], texture [10], shape [11–13], and attention [14]. These various and complex sensitivities are said to contribute to FG determination and the construction of perceptual organization [15, 16]. However, the neural mechanisms underlying FG segregation from natural scenes are not fully understood.

Natural images include rich local information, such as color, texture, and contour segments, which play crucial roles in FG segregation. Human observers are typically able to segregate figures and grounds in local natural image patches that lack global information [17, 18]. Recent investigations on FG organization in natural scenes have reported that local image features such as contour shapes [17, 19, 20] and spectral anisotropy [21] are crucial factors for FG segregation. Computational models based on surround modulation that pool only local information in natural images have been found to exhibit the capability for determining the direction of a figure along a border [22, 23]. A recent physiological study reported FG-dependent responses to local natural images in V4 neurons [24]. Investigations on intermediate-level visual areas with a focus on local information in natural scenes is a crucial step towards understanding the neural basis of FG segregation.

Our recent electrophysiological study reported that neurons in monkey V4 exhibit FG-dependent responses to natural image patches and their silhouettes [24]. Approximately one-fourth of the patch-responsive V4 neurons exhibited significant modulation of firing activity that was dependent on the positional relation between the figure region of the stimulus and the classical receptive field (CRF) of the neuron but not on luminance contrast. However, the responses of individual neurons were not capable of consistent FG discrimination across a variety of natural patches so that activities of a few tens of neurons were needed to accomplish consistent discrimination. The neural responses depended on whether a figure was projected onto the CRF center, and thus what FG organization in and around the CRF evokes strong responses has not been clarified. Investigations of the spatial extents of figure and ground regions that evoke FG-dependent neural responses are crucial for clarifying whether these neurons indeed signal FG organization.

Spike triggered averaging (STA), often exchangeably called reverse correlation, has been widely applied for the analyses of the receptive field structure of neurons in early visual areas [25–27]. The spike triggered covariance (STC) method has also been applied to estimate nonlinear receptive fields [28, 29]. In traditional STA and STC, white noise stimuli, typically binary random dot stimuli, were presented to exclude bias due to the finite number of stimuli and their spatiotemporal correlation. Theoretically, this is based on the facts that autocorrelation of white noise is an impulse and that input-output cross-correlation is proportional to the unit impulse response [30]. More generally, STA and STC are constrained by spherical symmetry in stimulus structure and white noise is one example [31, 32]. Intuitively, white noise can be considered to include all possible images and to exclude bias based on the image characteristics of stimuli. Although the traditional STA and STC using white noise have been successfully applied in early visual areas, it has not been applied to higher visual areas. A prominent reason is that neurons in the intermediate- to high-level areas barely respond to whitened images (random dots); they respond to higher-order image characteristics that were often invariant to luminance contrast. For instance, V2 neurons exhibit border ownership-dependent responses independent of luminance contrast [1], and V4 neurons exhibit FG-dependent responses independent of surface texture and contrast [24].

Since neurons in sensory areas, including intermediate- to high-level visual areas, responded vigorously to natural stimuli, which include much more diversity and complexity than traditional and artificial stimuli, a number of studies have performed spike triggered analyses using natural stimuli and reported incomplete but interpretable structures [33–36]. In spike triggered analyses with natural images, compensation for nonwhiteness has been proposed [34, 37–39]. However, this compensation is not applicable in estimating FG structures that evoke the neural response. The essence of the compensation is to reduce autocorrelation, which requires differentiation in space, diminishing the extent of the figure region and leaving only the borders between the figure and ground or the high-frequency components of (small) FG regions. This processing is fundamentally incompatible with the estimation of the *extent* of figure and ground regions. Furthermore, the randomization (whitening) of a figure region is not reasonable in terms of perception; the randomized regions no longer constitute *figures* in perception. Therefore, neither whitening of figures nor compensation of autocorrelation is reasonable, and thus the complete estimation of the receptive field structure with respect to FG is not possible. However, since figure shapes in natural images are diverse and their spatial correlation can be controlled, it is possible to evaluate what extent of figure and ground regions in and around their CRFs evoked neural responses.

We estimated the spatial extent of local figure and ground regions in natural images that evoked the FG-dependent responses in V4 neurons by applying the framework of STA. Although our previous study reported that the modulation of firing activity was dependent on the positional relation between the figure region of the stimulus and the CRF center of the neuron, the spatial extent of figure and ground regions that evoked strong responses have not been clarified. Our novel method combines neural responses and the organization of figure and ground regions that evoked these responses. Figure and ground regions in the images were assigned based on the human perception of the images (FG label) [17, 19, 24]. Weighted by the spike counts in response to the stimuli, the corresponding FG labels were averaged so that the regions responsive to FG (responsive field in response to figure and ground; RF-FG) were estimated. The proposed method is not capable of estimating the complete receptive fields in response to FG because our method relies on the complexity and diversity of natural images and does not assure whiteness in the FG labels. Furthermore, this method is not capable of estimating nonlinear interactions such as surround modulation and spatial invariance. However, our method is capable of estimating the best possible FG structure that evokes a strong response of the neuron for the set of presented stimuli. To evaluate the estimated structure, we compared it with the structure predicted for an ideal neuron that exhibits complete FG-dependent responses for the presented stimuli (ideal RF-FG). Since both RF-FGs were estimated based on FG labels with the same autocorrelation, this comparison cancels out the autocorrelation and excludes the bias due to nonwhiteness. To estimate the veridicality of the RF-FG estimated by our STA method (RF-FG_STA_), we also estimated the RF-FG with adaptive filtering (RF-FG_AF_) and compared the two. Adaptive filtering (AF) is a standard machine learning technique for estimating a best-fit linear filter without constraints on image structures such as spherical symmetry [40]. AF has been used to estimate the receptive fields of neurons in early visual areas and has successfully revealed the spatial structure of the receptive fields in agreement with those estimated by STA [35]. Furthermore, we estimated RF-FG based on STC (RF-FG_STC_) to evaluate the contribution of nonlinearity. Approximately 50% of the examined neurons showed a significant RF-FG_STA_, and most of them exhibited good similarities with the ideal RF-FG_STA_ and the RF-FG_AF_, indicating that the neuronal responses indeed depended on the FG configuration in and around their CRFs. The model responses based on the RF-FG_STC_ did not show a significant difference from those based on the RF-FG_STA_, suggesting a negligible contribution of nonlinearities such as spatial invariance of figure regions. The result appears meaningful for understanding the responses that underlie the segregation of figures and the construction of surfaces in natural scenes.

## Materials and methods

We analyzed the previously recorded and published neural activities [24] of three hemispheres of two female macaque monkeys (*Macaca fuscata*). The recorded data were available at [41]. All animal experiments were performed in accordance with the guidelines of the National Institute of Health (1996) and the Japan Neuroscience Society and were approved by the Osaka University Animal Experiment Committee (certification no: FBS-13-003). In short, animal surgeries were performed under full anesthesia (1–3% isoflurane, 70% N2O, 30% O2) through an intratracheal cannula. Vitals were monitored during surgery. An antibiotic and an anti-inflammatory and analgesic agent were administrated immediately after the surgery until a week later. As a preparation for neural recordings, the animals were anesthetized by the inhalation of 1–3% isoflurane in nitrous oxide through an intra-tracheal cannula. Yamane *et al*. [24] included human psychophysical experiments where all experiments were performed in accordance with the guidelines of the Japanese Psychological Association and the Code of Ethics of the World Medical Association (Declaration of Helsinki), and they were approved by the Research Ethics Committee of the Faculty of Engineering, Information, and Systems at the University of Tsukuba (certification number: 2014R52-2). Written informed consent was obtained from all participants prior to the psychophysical experiment. The details of the animal welfare and preparation, recording, visual stimuli, experimental design, and other aspects of procedures were previously described by Yamane *et al*. [24]; essential information of the experiments was summarized in this section.

### Visual stimuli

All stimuli used in the present study were identical to those used in the previous study [24]. This section summarizes the essential information of the stimuli. Refer to Yamane *et al*. [24] for details.

#### Natural image patches

**Fig 1:**
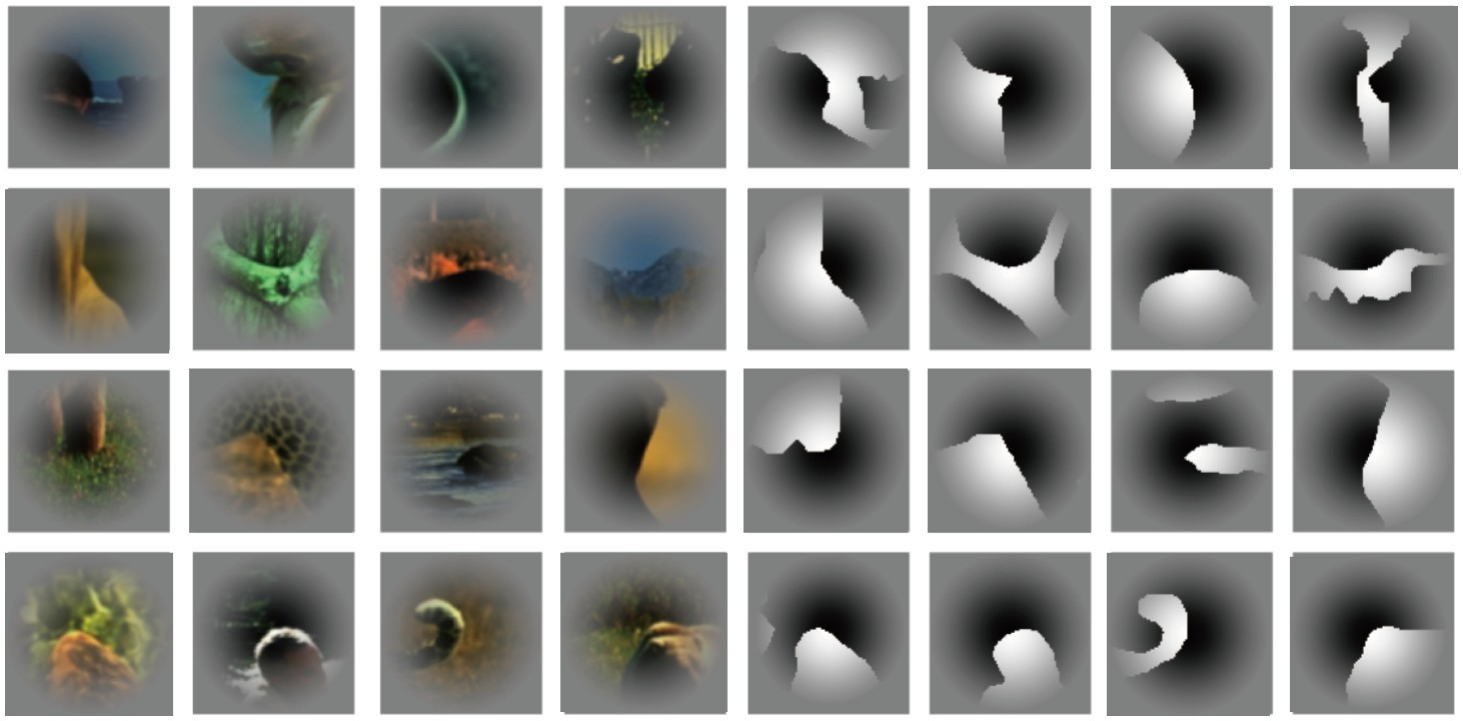
Examples of the natural and filled stimuli.

Natural image contours were drawn from the Human Marked Contours (HMC) available in the Berkeley Segmentation Dataset (https://www2.eecs.berkeley.edu/Research/Projects/CS/vision/grouping/fg/) [19]. A total of 105 subregions (69 × 69 pixels) were selected from the HMC that included the contours passing through the center of the patches. As the distribution of contour curvatures is highly nonuniform in natural scenes, the distributions of the degree of convexity, closure, and symmetry of contours were controlled such that the subregions were uniformly selected from each range of these characteristics [17]. Several examples are shown in Fig 1, and all the patches are shown in the Supplement, S1 Fig. The mirror image with respect to the tangent of the border passing through the patch center was prepared. The color of the mirror images was inverted so that the polarity of the color contrast remained constant with respect to the central border. The total number of patches of this set was 210. Although the patches are a small part of a full image, a few patches contained contextual information, such as half of a human face or a tail of a cat (refer to S1 Fig). These patches were not excluded from the stimulus set because no objective criterion for context was established and the number of them was a few (8). Since the extent of the patches that corresponded to the visual angle was approximately one tenth of the original full images, the probability of the appearance of context was much smaller in the patches than in the full images. The standard deviation of the spatial distribution of luminance over all natural-patches was 7% of the mean luminance, indicating that the average patch was close to a uniform mid-gray. The contrast towards the periphery was attenuated with a Gaussian function to obscure the boundary between the patch and the gray background. The veridical FG labels were previously determined by human psychophysical experiments [17, 24, 42]. Perceptual evaluations of figures and grounds in these natural patches were not substantially consistent across participants and trials, as reported in previous studies [19]. The mean perceptual consistency across all natural patches was 0.69, with a standard deviation of 0.11 (refer to [24] for details). The results indicate a fairly wide variety of perceptual consistency in FG evaluation across natural patches.

#### Filled patches

The natural image patches described above were filled with black on one side and white on the other side. Examples are shown in Fig 1, and all the patches are shown in the Supplement, S1 Fig. Including variations in contrast (2) and mirror image (2), the total number of stimuli in this set was 420. These four variations were pooled for analyses. The standard deviation of the spatial distribution of luminance over all filled patches was 5% of the mean luminance, indicating that the average patch over the filled patches was close to a uniform mid-gray. The veridical FG labels were determined by human psychophysical experiments [17, 24, 42]. The mean perceptual consistency across all filled patches was 0.77 with a standard deviation of 0.14 (refer to [24] for details), indicating fairly solid evaluations in FG determination to a similar but slightly higher degree than that across the natural patches.

**Fig 2:**
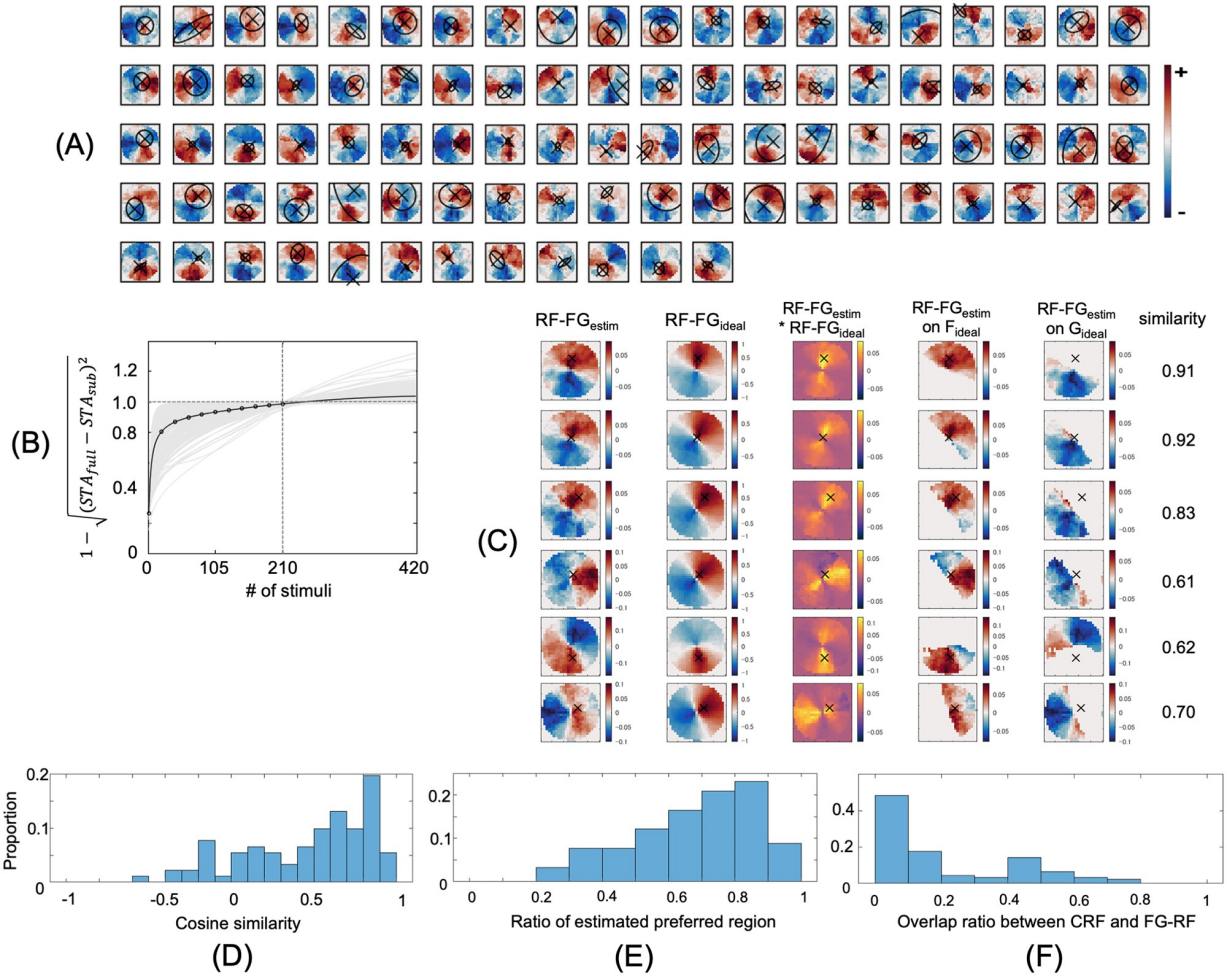
Estimated RF-FG_STA_s computed from the filled stimuli. (A) Estimated RF-FG_STA_s of the neurons that showed the significance in magnitude and a high convergence ratio (>0.9). The crosses and ellipsoids indicate the center and extent (1SD) of the CRF. The reddish and blueish colors indicate the regions that respond to figure (+) and ground (–), respectively, with the deeper colors representing the greater magnitudes. The magnitudes were normalized by the maximum. (B) Convergence of RF-FG_STA_ magnitude. The light and dark lines indicate the convergence of individual neurons and their mean, respectively, which were fitted by Eq (2). The ordinate is normalized so that the unity indicates the magnitude of RF-FG_STA_ with 210 stimuli. (C) Comparison between the estimated and ideal RF-FGs for six example neurons with preference to figure. The two left-most columns show the estimated and ideal RF-FG_STA_s, and the third column shows the product of the two. The fourth and fifth columns show the trimmed RF-FG_STA_s that were located on the figure and ground subregions of the ideal RF-FG_STA_, respectively. The magnitudes were normalized for each neuron, and the colors were normalized for each panel. The extent of panels was identical to that of stimulus patches. The right-most column indicates the cosine similarity between the estimated and ideal RF-FG_STA_s. The cosine similarity was given by the Euclidean dot product with two RF-FGs treated as vectors. (D) Distribution of the cosine similarity. (E) Distribution of the ratios between the extent of the estimated preferred-region and that of the ideal preferred-region. (F) Distribution of the overlap ratios between the extents of the CRFs and preferred regions.

Although the STA requires whiteness in the stimuli, our patch stimuli were not whitened; rather, our method relied on the variety of FG organization in natural images. The validity of the patch stimuli was computationally evaluated by comparing the RF-FGs computed from the filled stimuli and the dot stimuli that approximate white noise. The details of the model are given in Supplement, S2 Fig. The figure- and ground-preferring subregions in the model were predetermined by the RF-FG measured by the STA from the recorded data (refer to Fig 2(A)). We examined whether the predetermined RF-FGs were correctly estimated from the filled stimuli and the corresponding FG labels by the proposed STA method through the simulations of the model. Stimuli were either the filled stimuli that were used in our experiments or randomly placed single dots (1×1 pixel) with either -1 or +1 (representing figure and ground, respectively). We carried out STA and compared the RF-FGs computed from the filled and dot stimuli (RF-FG*s). Note that this model simulation aimed to examine the validity of pseudo-whiteness in the stimuli but not to propose neural mechanism underlying FG processing. The results of simulations showed that the median of cosine similarities between the RF-FG*s computed from the filled and dot stimuli was 0.87 (mean = 0.83, SD = 0.11) across the examined neurons, indicating good validity of the patch stimuli. Intuitively, a prominent difference between the patch and dot stimuli was the spatial extent that affected the spatial resolution in STA. For instance, RF-FG*s computed from the filled stimuli appear to be blurred in periphery compared to those computed from the dot stimuli. The cosine similarity for a small subregion whose extent was half of the typical CRF extent was 0.83, indicating the overall validity of the patch stimuli. The results of example model cells and the distribution of the similarities are shown in Supplement, S3 Fig.

### Design of the electrophysiological experiment

The color of the filled patches was chosen so that the maximum neural responses were obtained, whereas the color of the natural patches was not changed. Stimuli were also scaled to cover the CRFs of the recording units, more than three times larger than the rough estimate of the CRF diameter, yielding a stimulus size between 2.5 and 21 degrees. This scale was selected to ascertain the recordings from multiple neurons with their CRFs covering different regions of a stimulus with reasonable overlap. Stimuli were shown against a plain gray background on a 27-inch LCD monitor (CG275W Eizo; refresh rate, 60 Hz; white luminance, 125 cd/m^2^; black luminance: 1.3 cd/m^2^) placed at a distance of 57 cm from the monkey’s eye. All stimulus presentations were repeated 10 times within a session in a pseudorandom order and shown for 200 ms with a blank 200-ms interstimulus interval. The square-wave grating patches were included to determine the CRF center and the extent of individual neurons. The grating patches were presented at one of 25 positions in a 5x5 grid across the stimulus extent, without fine tuning for the recorded neurons. Thirty-two-channel silicon probes arranged linearly (A1X32-10 mm 50–413, A1X32-10 mm 100–413) or probes with eight shafts (A8X1 tetrode-2 mm 200–312) (Neuronexus Technologies, Ann Arbor, MI, USA) were used for recordings of the neural activity of V4 neurons. Collected neural signals were amplified (1000×), filtered (0.5–8 kHz), and sampled at 20 kHz. For the main analyses, single-unit spiking activities were sorted offline for each session. Refer to Yamane *et al*. [24] for details of the physiological experiment.

### Data analysis

For the examination of responsiveness to stimuli, we compared the firing rates of isolated single units during the prestimulus period (100–0 ms before stimulus onset) with those of the stimulus period (40–200 ms after stimulus onset) for all stimuli with t-tests or Welch’s t-test if equal variance was violated. A value of p < 0.05 was used as the criterion for responsiveness. To estimate the retinotopic location and extent of CRFs, we counted the number of spiking events in isolated single units during the presentations of the grating patches shown at different retinal positions. The center and extent of the CRFs were estimated from the mean spike count maps fitted by a two-dimensional Gaussian function. Based on the positional relation between the CRF center and the content of the image patch, we classified the patches for each neuron into two categories: the CRF center on the figure or on the ground (FG; “figure” or “ground”).

### Estimation of subregions responsive to figure and ground (RF-FG) by STA

Applying the framework of STA, we estimated the spatial structures of local figure-ground organization in the stimuli that evoked FG-dependent responses. We proposed combining the neural responses and the figure and ground regions within the presented stimuli. Figure and ground regions in the stimuli were assigned based on the human perception of the stimuli [17, 19, 24] and were tagged with +1 and -1, respectively (FG label). Weighted by the spike counts observed in response to the stimuli, the corresponding FG labels were averaged so that the regions responsive to FG (responsive field in response to figure and ground; RF-FG) were estimated. Hereafter, the terms RF-FG and *kernel* are used interchangeably. The RF-FG by STA is given by

$$RF-FG_{STA}=\frac{\sum_{i}^{N}FGlabel_{i}*spike_{i}}{\sum_{i}^{N}{\text{spike}}_{i}}-\frac{\sum_{i}^{N}FGlabel_{i}}{N},$$ (1)

where *spike* and *FGlabel* indicate the number of spikes in response to stimulus *i* and the corresponding FG label, respectively. *N* indicates the total number of FG labels (*N* = 210). For filled stimuli, the contrast polarity of the figure (black or white) was disregarded, and the same FG label was used for the pair of filled stimuli with opposite contrasts, adding the responses of the pair so that N = 210 for the filled patches. RF-FGs were expected to show the independence to image features including contrast and orientation since the responses to both contrast polarities and a wide variety of contours across stimuli were added into the computation. This processing was expected to extract the responses to FG and cancel out the responsiveness to other image features. The average across all FG labels was subtracted from the weighted average. This compensation for the nonuniformity of the FG labels was necessary since the present method used a limited number of structured FG labels without whitening. The mean FG label was equivalent to the RF-FG wherein the neural responses were equal across all FG structures, and thus, the subtraction of the mean FG label represents the cancellation of bias evoked by the nonuniformity of FG organization. The estimated STA ranged between –1 and +1, with positive and negative values indicating the preference to figure and ground regions, respectively; the greater values indicate the generation of the greater responses if a figure is projected onto the location. Intuitively, the RF-FG represents the best possible FG organization that evokes a strong response to the neuron but not any interaction such as surround suppression [43]. The significance of the kernel was estimated by the permutation wherein the spike counts for each stimulus were randomized. RF-FG_STA_ was considered significant if the magnitude (squared sum of all elements) of RF-FG_STA_ was significantly greater (p < 0.05) than the magnitudes of kernels with randomization (RF-FG*_STA_). The randomization was repeated 1000 times to obtain a set of RF-FG*_STA_s.

Since the number of stimuli was finite, it could be expected that the estimated RF-FG_STA_ did not reach convergence. We computed the magnitudes of RF-FG_STA_s with a limited number of FG labels in multiples of 20 and estimated the convergence ratio with respect to an infinite number of stimuli. The stimuli were randomly chosen every time, and the computation was repeated 100 times. To evaluate the convergence at N = 210, we fitted the data with a function [44]:

$$y=a{\left[1-{\left(\frac{b}{\text{exp}\left(\frac{x^{c}}{d}\right)-1+b}\right)}^{e}\right]}^{f},$$ (2)

where *a ~ f* are free parameters and optimized by the *fmincon* function in MATLAB [45]. The value of *a* was considered the convergent point at infinity. The ratio of convergence is given by the value of *y* at N = 210 divided by *a*. The RF-FGs with the ratio of 0.9 or greater were considered effective and subject to analysis. The relatively high ratio was chosen because cross-validation was not performed since the number of stimuli was limited. The mean number of spikes for estimating a single effective RF-FG was 876.

We defined a neuron as an ideal FG cell if it fired a single spike for all stimuli whose preferred region fell onto its CRF-center but did not fire for other stimuli. The RF-FG_STA_ of an ideal FG cell (the ideal RF-FG_STA_) is given by the mean of the FG labels whose preferred region fell onto its CRF-center. Since the border between figure and ground passes through the center of the patches in our stimulus set, the ideal RF-FG_STA_ shows an antagonistic structure with a preferred region on the CRF side and a non-preferred region on the other side with respect to the patch center. On the other hand, the RF-FG_STA_ of a non-FG cell that does not evoke FG-dependent responses is given by the mean of all FG labels, which is flat at zero, as seen from Eq 1. Actual RF-FG_STA_s tend to fall between these two extremes, and the similarity between the ideal and estimated RF-FG_STA_s is expected to indicate the degree of FG dependence in the responses. Note that the spatial autocorrelation of the FG labels provides the distribution of the FG extents.

### Estimation of linear RF-FGs by AF

AF is a standard machine learning technique for estimating a best-fit linear filter without constraints on image structures such as spherical symmetry [40]. In short, AF gradually searches for the best-fit receptive field as the number of trials increases, whereas STA deterministically estimates receptive fields directly from the stimuli and corresponding spike counts. In contrast to kernels estimated by STA using white noise, kernels estimated by AF can be dissociated from the veridical receptive field structure. It is expected to perform both methods in the evaluation of the receptive field structure if spherical symmetry is not assured; a similarity between the STA and AF kernels likely indicates veridicality. Conventional AF also depends on the stimulus luminance, similar to traditional STA; therefore, conventional AF is not directly applicable in estimating higher-order properties other than luminance, such as border ownership- and FG-dependent responses.

Recent studies have proposed AF based on the recursive least-square (RLS) algorithm for the estimation of RF structures [35]. We used the RLS algorithm to obtain the optimal linear kernels with respect to FG (RF-FG_AF_). An outline of the algorithm is provided in the Supplement (S4 Fig). The algorithm sequentially takes a pair of FG labels and the corresponding spike rate. The pixelwise product (Hadamard product) between the FG label and kernel, which represents the simulated response, is then taken. The algorithm sequentially modifies the kernel to minimize the difference between the Hadamard product and neural response. The number of input pairs was 4200 (210 stimuli × 2 contrasts × 10 trials) for the filled patches. Since the number of pairs was 2100 (210 stimuli × 10 trials) for the natural patches, we duplicated the pairs to obtain 4200 pairs. The presentation order of input pairs was randomized such that every consecutive stimulus was different.

### Estimation of nonlinear RF-FGs by STC

The STC is capable of estimating nonlinear RFs [28, 29, 32, 46]. The STC estimates RFs based on the spike-weighted covariance of stimuli, although the computational methods varied across the previous studies. An illustration of the concept around STA and STC is shown in the Supplement, S8 Fig. RF-FG by STC is given by [32]

$$\begin{array}{ll} RF-FG_{STC} & =C_{spike}-C_{base}\\ & =\frac{1}{N-1}\sum_{i=1}^{N}\left(FGlabel_{i}*spike_{i}-RF-FG_{STA}\right){\left(FGlabel_{i}*spike_{i}-RF-FG_{STA}\right)}^{t}\\ & -\frac{1}{N-1}\sum_{i=1}^{N}\left(FGlabel_{i}-RF-FG_{STA}\right){\left({\text{FGlabel}}_{i}-RF-FG_{STA}\right)}^{t} \end{array},$$ (3)

where *FGlabel* and *RF-FG*_*STA*_ are row vectors with a length of 625 (25 × 25), and *N* indicates the total number of FG labels (N = 210). *Spike*_*i*_ represents the normalized number of spikes for stimulus *i*. RF-FG_STA_ was subtracted because the distribution of the stimuli that evoked responses was biased from the distribution of the original stimuli. Although conventional STC assumes spherical covariance in stimuli similar to traditional STA, our method does not include whitening since it does not meet the aim of estimating RF-FG as discussed above; therefore, the estimated kernel does not provide complete subregions. We performed a compensation of subtracting the baseline that is covariance among FG labels, similar to the estimation of FG-RF_STA_. The filled stimuli had contrast-reversed pairs with identical FG labels. The responses to both contrasts were summed for the computation so that RF-FG_STC_s were contrast invariant.

Since the resolution was 25×25, we obtained 625 eigenvectors (RF-FG_STC_s), many of which do not represent meaningful kernels. As reported previously, the determination of significance is important but not straightforward [34, 46]. In the present study, effective RF-FG_STC_s were selected based on the significance of their magnitudes. The effectiveness of a kernel was defined based on the eigenvalues obtained from the randomized spike trains (RF-FG*_STC_). An eigenvalue represents the contribution of the eigenvector (such as RF-FG_STC_ and RF-FG*_STC_); therefore, the eigenvalues of RF-FG_STC_s that exceeded those of RF-FG*_STC_ were considered significant [34]. Specifically, RF-FG_STC_ was considered significant if two conditions were met: (1) its eigenvalue exceeded ±1 SD from the mean of the differences of the eigenvalues between the consecutive RF-FG*_STC_s along the rank of their eigenvalues, and (2) if the RF-FG_STC_ at the nearest neighbor along the rank in the ascendent (descendent) direction was also significant when its eigenvalue was positive (negative) (refer to Fig 7). These criteria were selected according to the STC analysis of V1 complex cells [34].

### STA and STA+STC model

We constructed computational models of individual neurons based on RF-FG_STA_ and RF-FG_STC_ and evaluated the effectiveness of RF-FG_STC_. In the STA model, the FG label corresponding to the presented stimulus was multiplied pixelwise with the RF-FG_STA_ and passed through rectification. The response of the STA model is given by

$$r_{STA,i}=w_{\text{STA}}*\left(FGlabel_{i}*RF-FG_{STA}\right),$$

$$r_{STA,i}^{+}=\left\{\begin{array}{rr} r_{STA,i}, & r_{\text{STC}}>0\\ 0, & r_{\text{STC}}\leq 0 \end{array}\right.,$$

where *w*_STA_ indicates the weight to be optimized to minimize the root-mean-square (RMS) error between the model responses and the corresponding neural responses. The architecture of the STA+STC model is illustrated in Fig 9(A). The FG label corresponding to the presented stimulus was multiplied pixelwise with the RF-FG_STA_ and RF-FG_STC_s, and then the products were added/subtracted and passed through rectification. The STA+STC model is given by

$$r_{STA+STC,i}=r_{STA,i}+\underset{j}{\sum }w_{STC^{+},j}*\left|FGlabel_{i}*RF-FG_{STC^{+},j}\right|-\underset{k}{\sum }w_{STC^{-},k}*\left|FGlabel_{i}*RF-FG_{STC^{-},k}\right|$$

$$r_{STA+STC,i}^{+}=\left\{\begin{array}{rr} r_{STA+STC,i}, & if>0\\ 0, & if\leq 0 \end{array}\right.,$$

where $RF-FG_{STC^{+},j}\;(RF-FG_{STC^{-},k})$ indicates the *j-*th (*k*-th) effective STC kernel with positive (negative) eigenvalues and where $w_{{STC}^{+},j}$ indicates the weight for the corresponding kernel. RF-FG_STC_s modulate the response of RF-FG_STA_. Specifically, the products with RF-FG_STC+_s were added to the product with RF-FG_STA_ because RF-FG_STC+_s, which have positive eigenvalues, represent the variance in the direction where the variance is great. In contrast, the products with RF-FG_STC–_s, which have negative eigenvalues, were subtracted from the product with RF-FG_STA_ [46]. The weights were optimized to minimize the RMS error between the model responses and the corresponding neural responses. The initial value of *w*_*STA*_ was set to the optimal value obtained by the STA model, and those for *w*_*STC*_*s* were set to zero; therefore, the model was updated only if its root mean square error (RMSE) was lower than that of the STA model. During the optimization, the *w*_*STA*_ and *w*_*STC*_ were updated simultaneously.

### The overlap ratio between the extents of CRF and RF-FG

To quantify the overlap of the spatial extents of the CRF and the preferred region in RF-FG, we defined the overlap ratio as:

$$\text{Overlap}\;\text{ratio}=\frac{CRF\wedge RF-FG_{pref-region}}{CRF\vee RF–FG_{pref-region}}.$$

*CRF* and *RF-FG*_*pref-region*_ represent the spatial extents of the CRF and the preferred subregion of a neuron, respectively. The extent of the CRF was given by the standard deviation of the Gaussian that approximated the CRF extent. The extents of the figure and ground subregions were given by the regions with positive and negative values, respectively. The regions with zero values in RF-FG were excluded from the computation. The symbols ∧ and ∨ represent the logical AND and OR, respectively (the overlap between the two and the subtraction of the overlap from the summation of the two, respectively). This ratio takes one if the two extents are identical and completely overlapped to each other and zero if the two are not overlapped at all.

## Results

### Subregions responsive to FG estimated by STA

**Fig 3:**
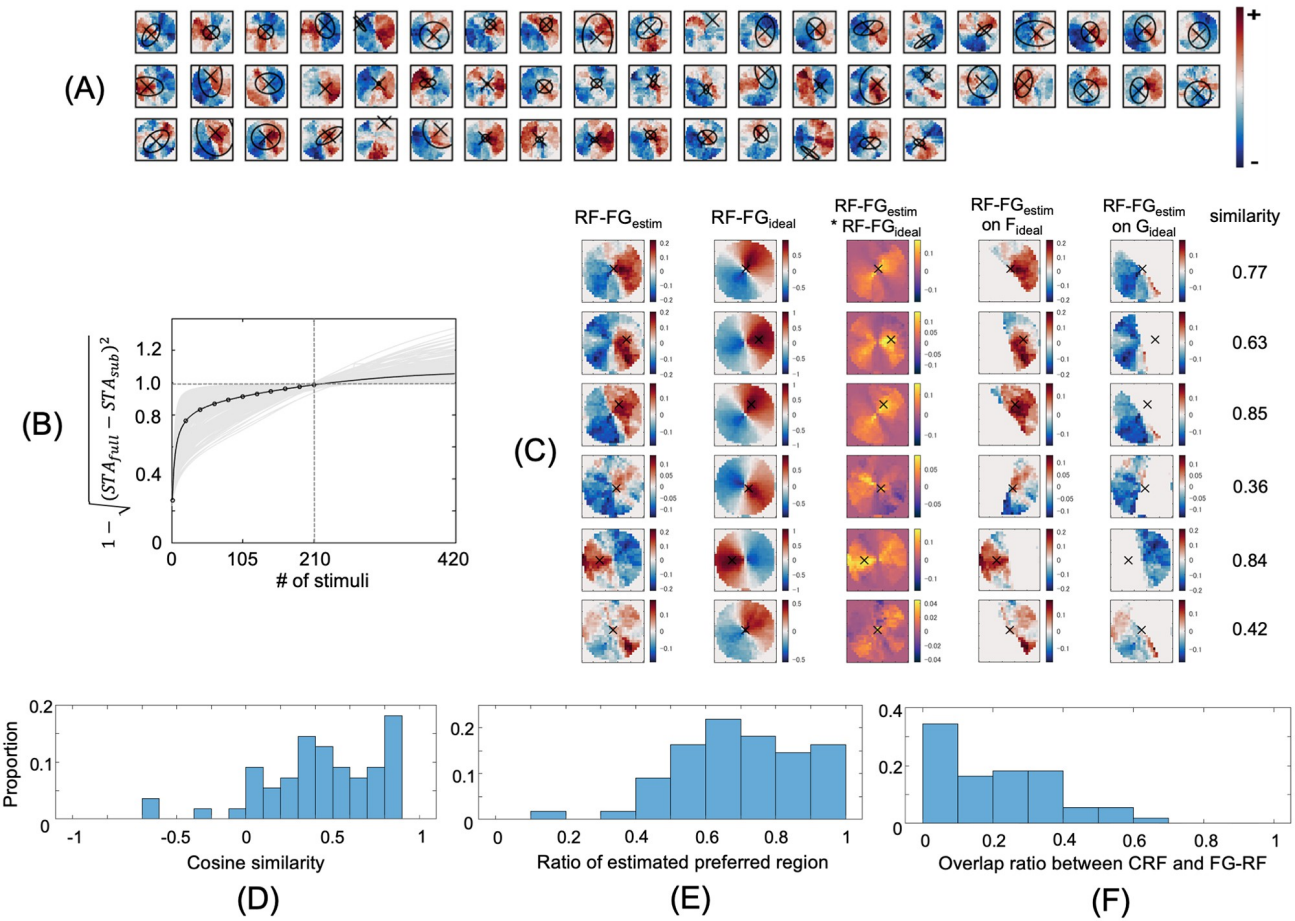
Estimated RF-FG_STA_s computed from the natural stimuli. The same conventions were used as in Fig 2. (A) Estimated RF-FG_STA_s. (B) Convergence of RF-FG_STA_ magnitude. (C) Comparison between the estimated and ideal RF-FGs for six example neurons with preference to figure. (D) Distribution of the cosine similarity. (E) Distribution of the ratio between the extents of the estimated preferred-region and that of the ideal preferred-region. (F) Distribution of the overlap ratios between the extents of the CRFs and preferred regions. The RF-FG_STA_s computed from the natural stimuli exhibited similar characteristics to the RF-FG_STA_s computed from the filled stimuli.

We estimated the spatial extent of local figure and ground regions in natural images and their silhouette images that evoked FG-dependent responses in V4 neurons. The stimulus set was designed to include a wide variety of contour shapes. The contours (boundaries between figure and ground) passed through the center of stimuli to include both figure and ground regions facing each other with respect to the center. We analyzed the spiking activities of neurons that were visually responsive and their CRF centers were located within the presented stimuli, with spikes observed 40–200 ms after stimulus onset. The RF-FG_STA_s were estimated based on STA; the FG labels corresponding to the stimuli were averaged with weights based on the spike rate generated by the stimulus. For filled stimuli, the contrast polarity of the figure (black or white) was disregarded, and the same FG label was used for the pair of filled stimuli with the opposite contrasts; therefore, the estimated RF-FG_STA_s were contrast independent. The independence to other image features was also expected since the stimuli included a wide variety of contours in the stimuli. For natural stimuli, the independence to color and texture was also expected. We selected effective RF-FG_STA_s based on the significance of magnitude and good convergence. The effective RF-FG_STA_s for the filled stimuli are shown in Fig 2(A). The RF-FG_STA_s for all examined neurons are provided in the Supplement (S5 Fig). Sixty-five percent (156/239) of neurons showed a significant magnitude in RF-FG_STA_ with respect to the kernels computed with randomization (p<0.05). The convergence of RF-FG_STA_ is shown in Fig 2(B), wherein 68% (162/239) of neurons showed good convergence (>0.9 at *N* = 210 with respect to an infinite number) for the filled stimuli. Among the neurons with good convergence, 57% (92/162) of neurons showed a significant magnitude of RF-FG_STA_. The proposed method determined effective (significant magnitude and good convergence) RF-FG_STA_ with the filled stimuli for 38% (92/239) of the examined neurons. With natural stimuli, 21% (55/265) of neurons showed effective kernels (Fig 3). When combining RF-FG_STA_s for filled and natural stimuli, approximately 50% (134/265) of neurons showed effective kernels. This result suggests that the neurons were capable of coding FG regions in natural stimuli, in agreement with the FG-dependent response reported previously [24]. In the following sections, we focus on the effective RF-FG_STA_s that showed significant magnitude and good convergence.

Most of the estimated RF-FG_STA_s exhibit a subregion responding to figures and another to the ground. These RF-FG_STA_s represent the best possible FG structure that evokes a strong response of the neuron for the set of presented stimuli. Our stimulus set was designed such that each stimulus included both figure and ground regions, with the boundaries between the two passing through the stimulus center, and were controlled to include a wide variety of shapes. Since these stimulus characteristics provide strong constraints on the estimation of FG structures, the computed structures need to be carefully evaluated. The present result was predicted if the neuron in fact responded vigorously to figure or ground but not to the other. Intuitively, a figure subregion matches the mean of figure regions if a neuron equally responded to all figures but not to grounds (an ideal F cell). In contrast, if a neuron responded to both figure and ground, no subregion emerges. Since the FG boundaries passed through the center of stimuli, a preferred subregion appears on the CRF side and a non-preferred subregion on the other side with respect to the center. The RF-FG of an ideal cell (ideal RF-FG) depends on its FG preference and the spatial location of the CRF center (refer to the Materials and methods for details). An agreement between the estimated and ideal RF-FGs indicates that the neurons indeed code the FG organization in stimuli. A comparison between the estimated and ideal RF-FGs is shown in Figs 2(C) and 3(C) for example neurons (refer to S6 Fig). The similarities between the estimated and ideal RF-FGs were widely distributed across the neurons with medians of 0.59 and 0.46 for filled and natural stimuli, respectively (Figs 2(D) and 3(D)). The inclusion of neurons with ineffective RF-FGs but with significant FG modulation determined by mean spike count (ANOVA, p<0.05) slightly increased the medians of similarities to 0.66 and 0.74 for filled and natural stimuli, respectively, suggesting that the criteria for effectiveness might have been stricter than necessary. Within ideal preferred regions, the extents of the estimated preferred regions were dominant over the non-preferred regions with medians of 0.73 and 0.70 across the neurons for filled and natural stimuli, respectively (Figs 2(E) and 3(E)). These results indicate that the neural responses depended on the FG organization in the presented stimuli.

The spatial extents of the subregions appear larger than those of the CRF (Figs 2(A) and 3(A)). The medians of the overlap ratios between the CRFs and preferred subregion were 0.11 and 0.20 for filled and natural stimuli, respectively (Figs 2(F) and 3(F)). The overlap ratio was defined such that it took one if the extents of the two were identical and completely overlapped, and zero if they were not overlapped at all (refer to Materials and methods). This result could indicate that surround modulation greatly contributed to the FG-dependent responses. However, the extents of CRFs were determined by grating stimuli that could be different from the optimal stimulus of the neuron. The veridical extents of the CRFs could be different if other visual attributes such as curvature, texture, and color were used for the determination of the CRF [16]. Further examinations are necessary to clarify the exact roles of the CRF and surrounding modulation.

**Fig 4:**
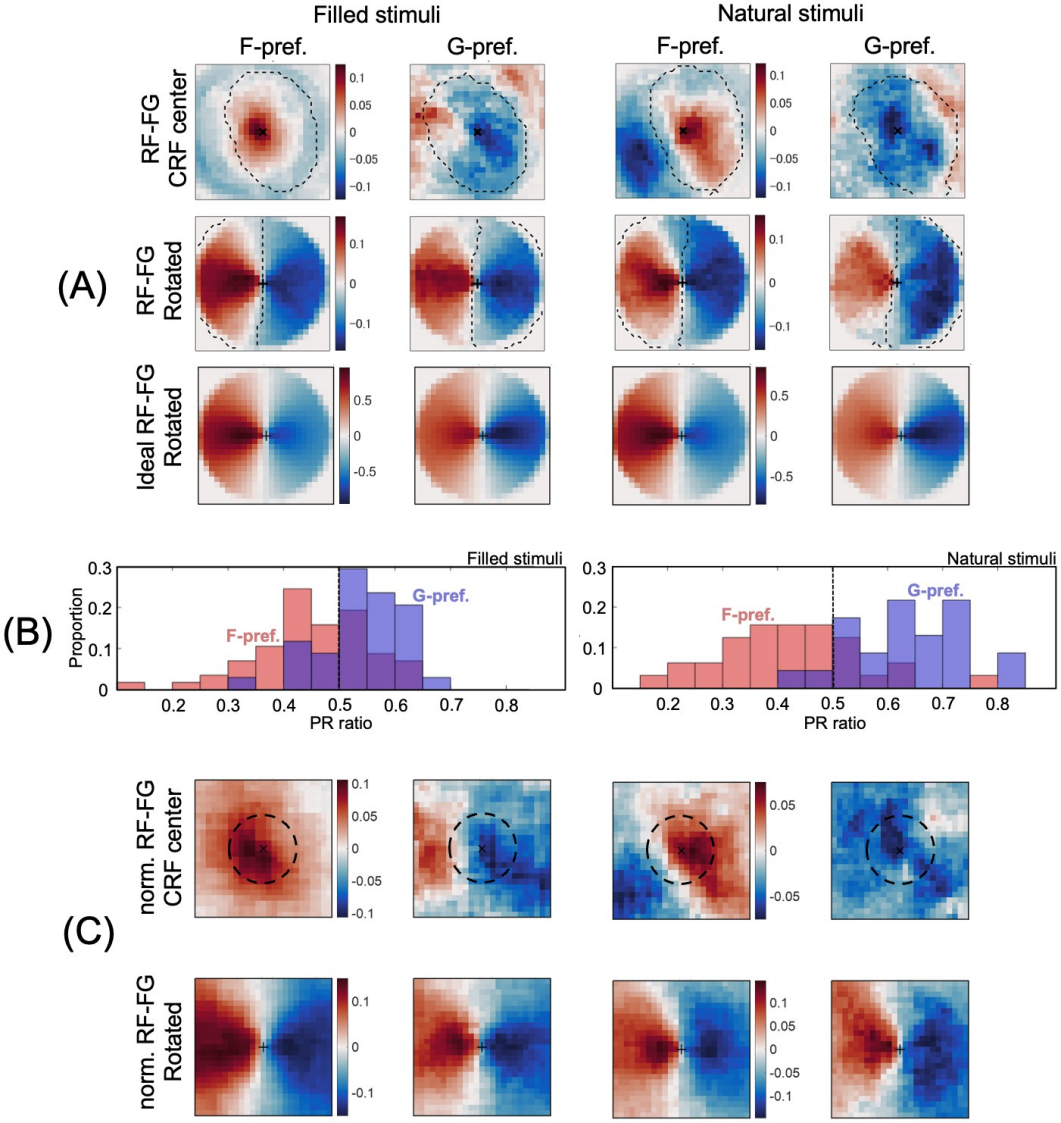
The mean RF-FG_STA_s across neurons. (A) The mean RF-FG_STA_s with aligned CRFs estimated from filled and natural stimuli (the left and right two columns, respectively). The top row shows the mean RF-FG_STA_s across the neurons. The left and right columns show the RF-FG_STA_s of the neurons that preferred figure and ground, respectively. The reddish and blueish colors indicate the regions that respond to figure (+) and ground (–), respectively, with the deeper colors representing the greater magnitudes. The preferred regions appear around the CRF center as indicated by the black cross at the panel center. The middle row shows the mean RF-FG_STA_s with rotation; individual RF-FG_STA_s were rotated with respect to the patch center to align the center of gravity of the figure and ground regions to the left and right, respectively. The magnitudes were normalized by the maximum value, and the colors were normalized for each panel. The extent of panels was identical to that of the stimulus patches. The dotted lines indicate the extent of the preferred region in the ideal RF-FG_STA_s. The bottom row shows the mean ideal RF-FG_STA_s with rotation. (B) The distribution of the extent of the preferred subregions in PR ratio. The red and blue colors indicate the ratios for figure- and ground-preferred neurons, respectively. (C) The mean RF-FG_STA_s with normalization (scaling) by the CRF extent. The dotted lines indicate a circle with the area equal to the mean area of the scaled CRFs. The same conventions were used as in panel (A).

The mean RF-FG_STA_s across neurons with aligned CRF centers are shown in Fig 4(A) together with the mean ideal RF-FG_STA_s. The neurons with figure preference show a figure subregion around the CRF center and a ground subregion in the periphery of the figure subregion. In contrast, the neurons with ground preference show a ground subregion around the CRF center and a figure subregion in the periphery. These subregions match those in the mean ideal RF-FG_STA_s, indicating that these neurons were indeed responsive to FG organization. The extents of preferred subregions for estimated RF-FG_STA_s appear smaller and larger for figure- and ground-preferred neurons, respectively, than for ideal RF-FG_STA_s. Since FG boundaries always passed through the patch center, subregions are expected to have the opposite preferences to figure and ground with respect to the patch center (an antagonistic structure). To clarify the characteristics of the subregions including their antagonism, we rotated the RF-FG_STA_s of figure preferred neurons with respect to the patch center so that the center of gravity of a figure subregion came to the left (Fig 4(A)). Similarly, we rotated the RF-FG_STA_s of ground preferred neurons so that a ground subregion came to the right. We observed a clear antagonistic structure of figure and ground subregions with respect to the patch center. The ratios of the extents for preferred subregions with respect to that for non-zero regions (PR ratio) were 0.41 and 0.64 for figure- and ground-preferred neurons, respectively, for natural stimuli (0.42 and 0.57 for filled stimuli), while the PR ratio for ideal RF-FG_STA_s was 0.50. The extent of figure subregion for figure-preferred neurons was significantly smaller than that for ideal neurons (t-test, p<0.05; refer to Fig 4(B)). This tendency agrees with the Gestalt principle for a smaller figure. It also appears that the figure subregion tends to show convexity in the direction of ground, suggesting a preference for a convex figure.

To clarify the extent of subregions with respect to the CRFs, we also computed the mean RF-FG_STA_s across neurons scaled by the extent of their CRFs (Fig 4(C)). The normalized mean RF-FG_STA_s show that the tendency of a smaller figure-subregion continues to the outside of the CRF. Within an extent twice as large as the CRF, the PR ratios were 0.43 and 0.61 for figure- and ground-preferred neurons, respectively, with natural stimuli (0.45 and 0.56 with filled stimuli). The convexity of the figure subregion appears clearer than in RF-FG_STA_s without normalization. These results indicate that the FG-dependent responses in V4 neurons are in fact dependent on the FG configuration in and around their CRFs.

**Fig 5:**
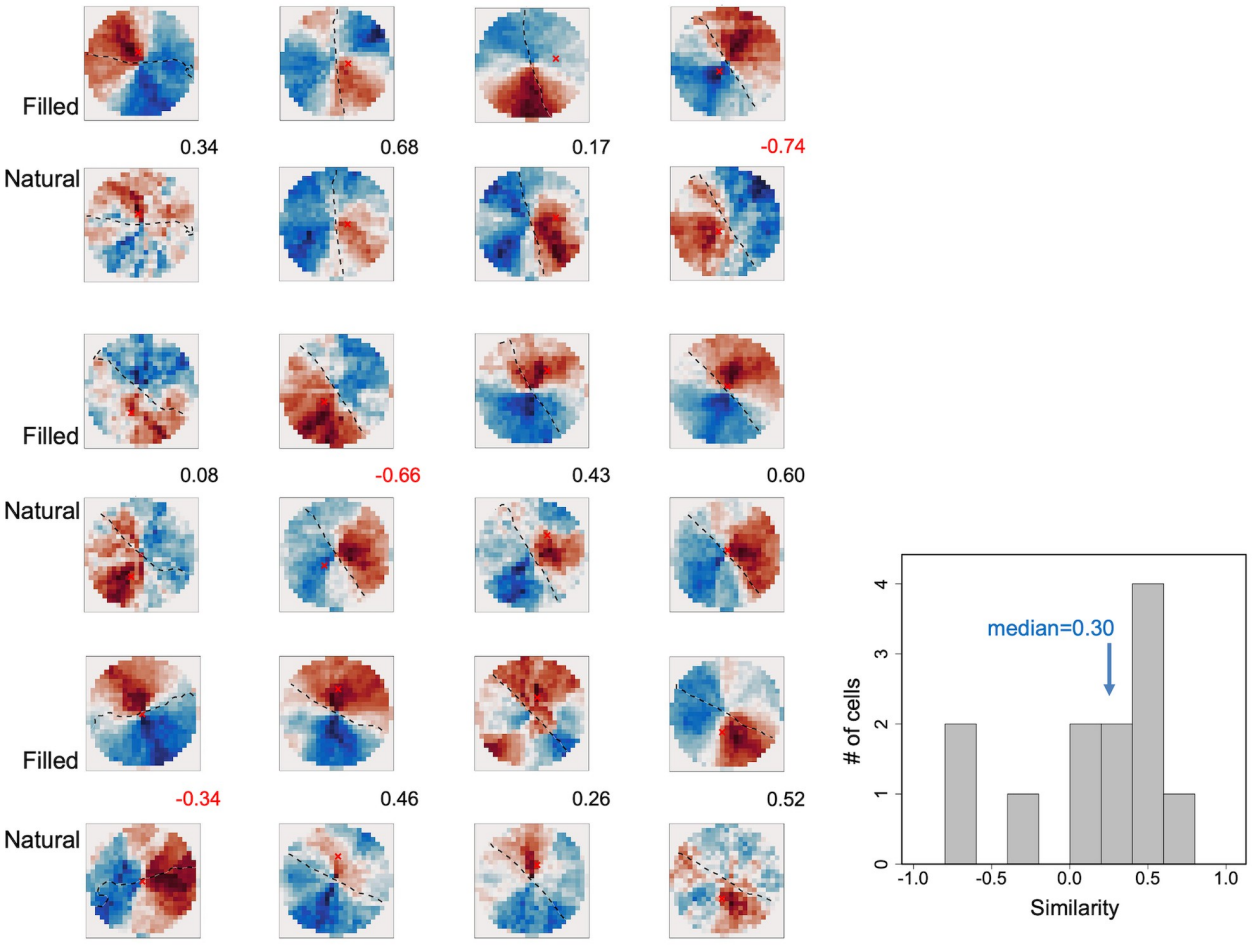
Similarity between RF-FGs estimated from filled and natural stimuli. The estimated RF-FG_STA_s of twelve neurons that responded to both natural and filled stimuli (in odd and even numbered rows, respectively). The same conventions were used as Fig 2. The values between the panels indicate the cosine similarity between the RF-FG_STA_s estimated from filled and natural stimuli. The magnitudes were normalized for each neuron. The reddish and blueish colors indicate figure- and ground-subregions, respectively, with the deeper colors representing the greater magnitudes. The right panel shows the distribution of the similarity.

The estimated RF-FGs were contrast independent since filled stimuli consisted of pairs of opposite contrast polarities (a pair of black and white figures with identical contours) and natural stimuli consisted of a variety of contrasts. To further examine the cue independence of the RF-FGs, we computed the similarity between those estimated from filled and natural stimuli. We examined twelve FG-effective neurons that were responsive to both natural and filled stimuli. Nine neurons showed positive correlations, and the median across all twelve neurons was 0.30 (Fig 5), suggesting substantial cue invariance between filled and natural stimuli.

### RF-FGs estimated by AF

**Fig 6:**
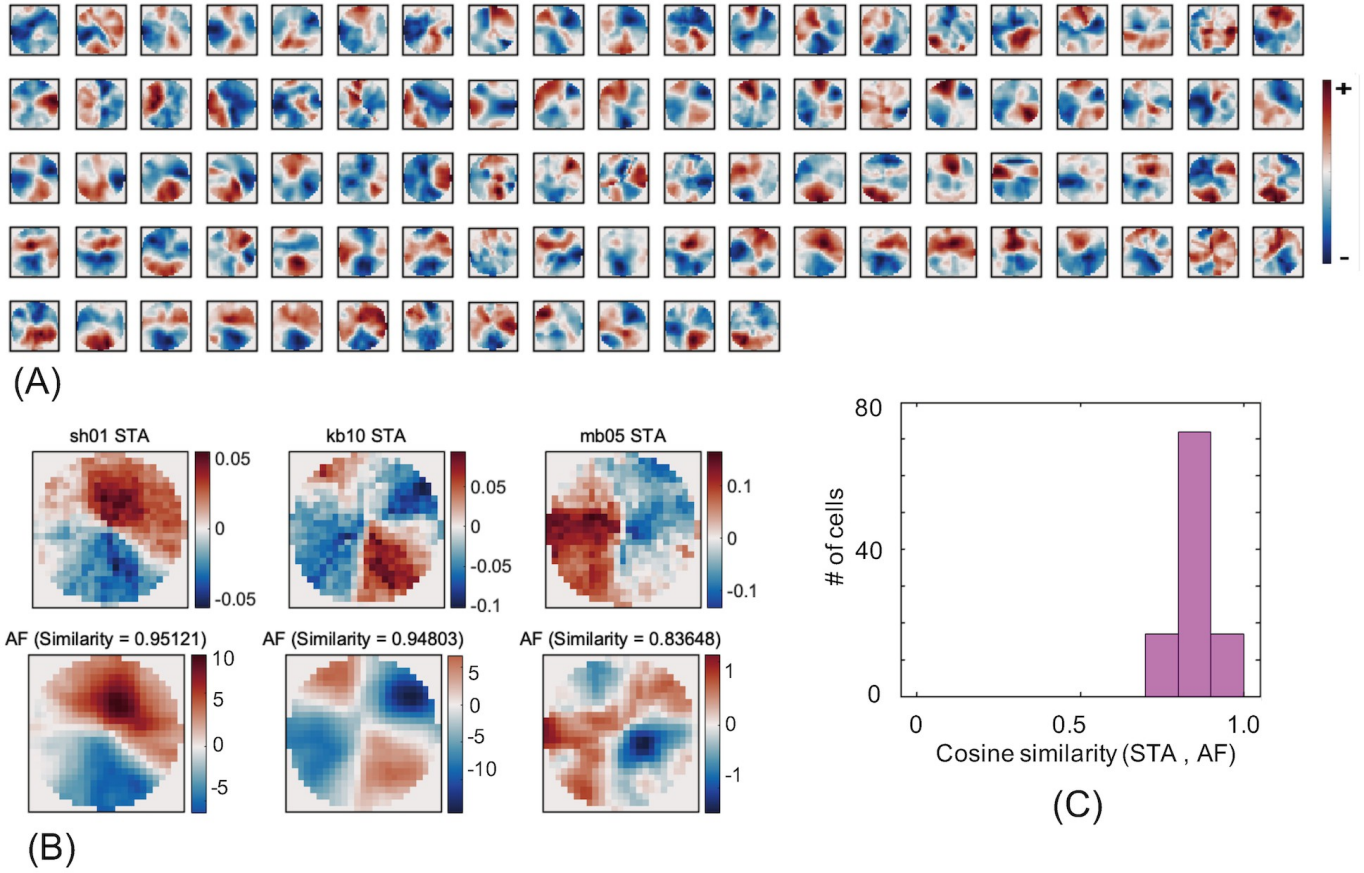
Estimated RF-FG_AF_s computed from the filled stimuli. (A) Estimated RF-FG_AF_s of the neurons that were shown in Fig 2. The same conventions were used as in Fig 2. (B) RF-FG_STA_s (top) and RF-FG_AF_s (bottom) of three example neurons with the cosine similarity between the two. Reddish and blueish colors indicate the regions that respond to figure and ground, respectively. Note that the magnitudes of RF-FG_STA_ and RF-FG_AF_ cannot be compared directly since RF-FG_STA_ corresponds to the normalized bias towards figure and ground regions (positive and negative, respectively) while RF-FG_AF_ corresponds to the spike rate (Hz) when multiplied with a stimulus and rectified. (C) Distribution of the cosine similarity between the normalized RF-FG_STA_ and RF-FG_AF_. The RF-FG_STA_ and RF-FG_AF_ show characteristics similar to each other.

Recent studies have proposed AF based on the RLS [35] that does not require any strong assumption, such as whiteness in stimuli. We estimated RF-FG based on AF (RF-FG_AF_) with the aim of evaluating the structure of RF-FG_STA_. The similarity between RF-FG_AF_ and RF-FG_STA_ supports the validity of the structure of estimated subregions. The estimated RF-FG_AF_s of the neurons with effective RF-FG_STA_s are shown in Fig 6(A) for the filled stimuli. A number of RF-FG_AF_s show similar structures to RF-FG_STA_s, with subregions responding to the figure and ground. For close visual inspection between the RF-FG_AF_ and RF-FG_STA_, the kernels of three example neurons are shown in Fig 6(B). To quantitatively clarify the similarity between RF-FG_AF_ and RF-FG_STA_, we computed the cosine similarity between the two. The mean similarity across the effective neurons was 0.85, and the SD was 0.05, indicating good similarity between the two (Fig 6(C)). The RF-FG_AF_ for the natural stimuli exhibited similar characteristics (mean = 0.85, SD = 0.03; refer to Supplement, S7 Fig). The similarity between the RF-FG_AF_ and RF-FG_STA_ suggests the validity of the estimated subregions responsive to figures and grounds.

### Nonlinear RF-FG based on STC

**Fig 7:**
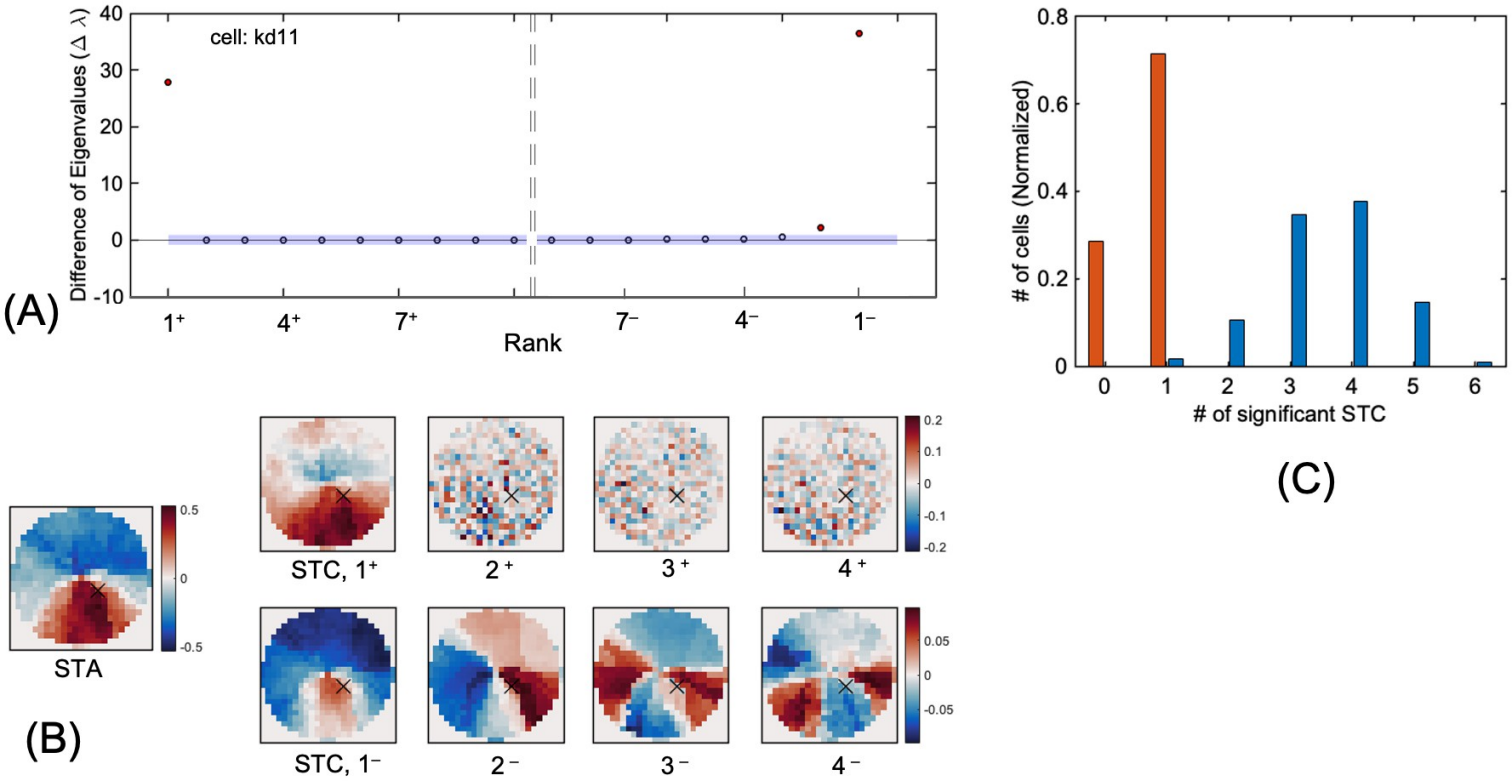
Computation of RF-FG_STC_s. (A) Difference of eigenvalue in the rank order of eigenvalues for an example neuron. Rank of +1 and –1 represent those with the largest (positive) and smallest (negative) eigenvalues, respectively. The largest 10 and smallest 9 eigenvalues were shown here. Blue error shade indicates ±1 SD of the difference in eigenvalues estimated from randomization. Red dots indicate significance. Three RF-FG_STC_s were considered significant (1^+^, 1^−^, and 2^−^). (B) RF-FG_STA_ and RF-FG_STC_s estimated for an example neuron. The top row indicates RF-FG_STC_s with the numbers corresponding to the rank in ascending order from the positive (1^+^, 2^+^, 3^+^, and 4^+^). The bottom row indicates RF-FG_STC_s with the numbers in descending order from the negative (1^−^, 2^−^, 3^−^, and 4^−^). Reddish and blueish colors indicate the polarity of the RF-FG_STC_. The same conventions were used as in Fig 2. The significant RF-FG_STC_s (1^+^, 1^−^, and 2^−^) show a clear dichotomy between figure and ground while nonsignificant RF-FG_STC_s show multiple regions (3^−^ and 4^−^) and mosaic-like distributions (2^+^, 3^+^, and 4^+^). (C) Distribution of the number of significant RF-FG_STC_s for single neurons. Red and blue bars indicate the RF-FG_STC_ counted from the largest (positive) and smallest (negative) eigenvalues, respectively. A number of neurons had a few significant RF-FG_STC_s with the median of three.

To examine the sufficiency of the linear kernels estimated by STA and AF, we estimated nonlinear RFs in response to FG regions based on STC (RF-FG_STC_). STA and AF estimate linear RFs based on the spike-weighted mean of stimuli. Alternatively, STC estimates RFs based on the spike-weighted covariance of stimuli. If multiple functional subregions coexisted and their mean was close to zero, STA might show no structure, but STC might reveal the subregions. An illustration of the concept of STA and STC is shown in the supplement, S8 Fig. We selected effective RF-FG_STC_s based on the significance of the magnitude [34]. Specifically, we evaluated the difference in eigenvalues along the rank order of eigenvalues and compared the magnitude of the difference with that evaluated from randomized spike trains (refer to the Materials and methods for details). The distribution of the difference in eigenvalues of an example neuron is shown in Fig 7(A) wherein the kernels of rank 1^+^, 1^−^, and 2^−^ were effective (1^+^ and 1^−^ represent the rank with the maximum (positive) and minimum (negative) eigenvalues, respectively). The estimated RF-FG_STC_s of the example neuron is shown in Fig 7(B) together with RF-FG_STA_. The effective RF-FG_STC_ s (1^+^, 1^−^, and 2^−^) exhibit two subregions in response to the figure and ground. In contrast, ineffective kernels exhibit multiple subregions or mosaic-like structures. Approximately 85% of neurons had a small number (1~6) of effective RF-FG_STC_s, as shown in Fig 7(C).

**Fig 8:**
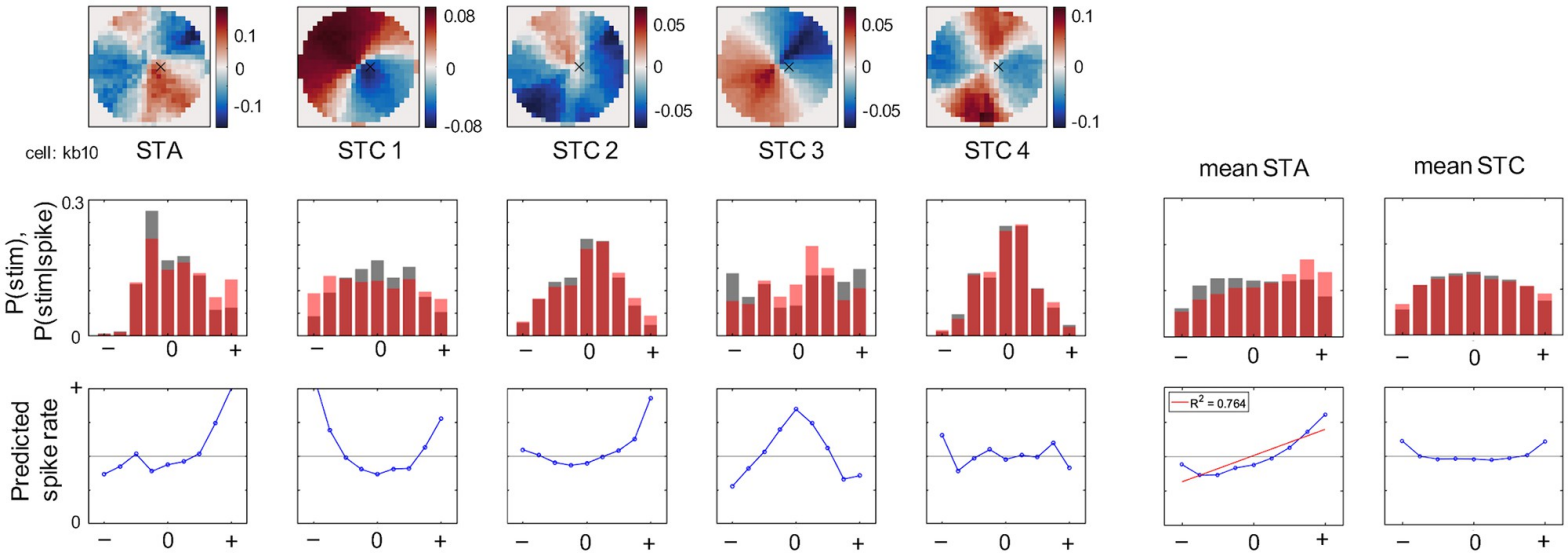
Estimated spike rates and the linearity of RF-FG_STA_ and RF-FG_STC_s. The top row shows the estimated RF-FG_STA_ and RF-FG_STC_s for an example neuron. The same conventions were used as in Fig 2. The middle row shows the distributions of P(stimulus) and P(stimulus|spike) in gray and red bars, respectively. The classes indicate the stimuli in the rank order of the responses they evoked (the normalized inner-product between the RF-FG and stimulus). P(stimulus|spike)/P(stimulus) is proportional to P(spike|stimulus) that corresponds to the predicted spike rates which are shown in the bottom rows. The linearity of the spike rates indicates the linearity of the operation of the kernel. The right-most column and the second column from the right show the mean across the RF-FG_STC_s and RF-FG_STA_s of all examined neurons, respectively. The mean RF-FG_STA_ shows fair linearity with R^2^ = 0.764 (the regression line in red; p<0.001), while the RF-FG_STC_ does not.

The RF-FG_STA_s are expected to exhibit linearity, while the RF-FG_STC_ s are not. We confirmed whether the RF-FG_STA_s and RF-FG_STC_ s, in fact, exhibit linearity and nonlinearity, respectively, based on Bayesian theorem. In essence, the probability of spike rate when a stimulus is presented (P(spike|stimulus)) was proportional to P(stimulus|spike)/P(stimulus) [28]. These probability distributions of an example cell and the mean distributions across the RF-FG_STA_s and RF-FG_STC_s are shown in Fig 8. The probabilities are in the rank order of the pixelwise product between the kernel and stimulus; therefore, the abscissa represents the stimuli in the order of the predicted response magnitude evoked by the stimulus. The predicted spike rate for the RF-FG_STA_s shows linearity (R^2^ = 0.764 for the mean), while that for the RF-FG_STC_s does not (Fig 8 bottom). As expected, the RF-FG_STA_s appear to show linearity, while the RF-FG_STC_s do not. In the next section, we examine the contribution of the nonlinearity based on the estimated RF-FG_STC_s.

### Models based on STA and STC

**Fig 9:**
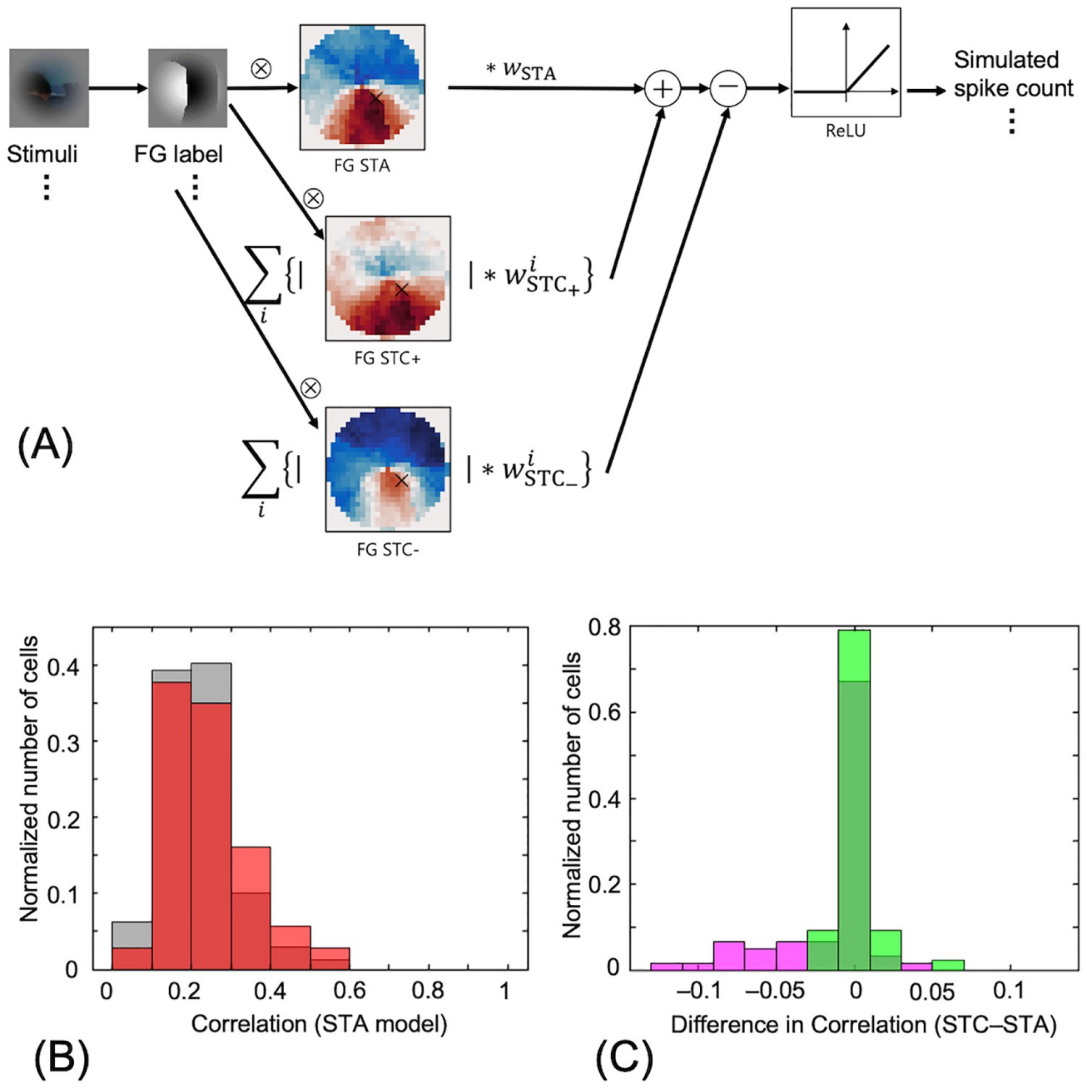
The model combining RF-FG_STA_ and RF-FG_STC_s. (A) Illustration of the model combining RF-FG_STA_ and RF-FG_STC_s. (B) Distribution of the correlation between the neural spike rate and the responses of the model with RF-FG_STA_ but without RF-FG_STC_ (STA model) in response to the corresponding filled stimuli. The gray and red bars indicate all examined neurons and the neurons with the good convergence and significant magnitude, respectively. (C) Distribution of the difference in the neural correlation between the STA model and STA+STC model. Red and green bars indicate those estimated for the filled and natural stimuli, respectively. The distribution of the difference between the STA and STA+STC models was centered around zero, indicating weak or no contribution of the RF-FG_STC_s.

To estimate the contribution of nonlinear processing (RF-FG_STC_) to FG-dependent responses, we constructed computational models of individual neurons based on RF-FG_STA_ and RF-FG_STC_ and evaluated the effectiveness of RF-FG_STC_. The architecture of the model is illustrated in Fig 9(A). The FG label corresponding to the presented stimulus was multiplied pixelwise with the RF-FG_STA_ and RF-FG_STC_s, and then the products were added/subtracted and passed through rectification. Within the characteristic space of stimuli, RF-FG_STA_ represents the distance and direction to the center of gravity among the stimuli to which the neuron responded. RF-FG_STC_s represent the bases (variance) for the distribution of the stimuli to which the neuron responded (refer to S8 Fig). Although the spike rate could be estimated by RF-FG_STA_ alone, the addition of RF-FG_STC_ would yield a better representation, as RF-FG_STC_ introduces the variance of the distribution. To modulate the RF-FG_STA_ response, the products with positive RF-FG_STC_s (those with positive eigenvalues) were added to the product with RF-FG_STA_, and the products with negative RF-FG_STC_s were subtracted [46]. The positive and negative RF-FG_STC_s have greater and smaller variance, respectively, in the stimulus space. Intuitively, a greater number of stimuli evoke responses with positive RF-FG_STC_s, and a smaller number of stimuli evoke responses with negative RF-FG_STC_s. Therefore, positive and negative RF-FG_STC_s can be considered to facilitate and suppress neural responses, respectively. The weights for RF-FGs were optimized to minimize the RMS error between model responses and the corresponding neural responses.

To evaluate the effectiveness of the RF-FG_STC_, we computed Pearson’s product correlation between the neural and model responses. Specifically, we computed the neural correlations of the model with both RF-FG_STA_ and RF-FG_STC_ (STA+STC model) and that of the model with RF-FG_STA_ alone (STA model) and compared the correlations between the two models. The free parameters of the model were optimized for each model (refer to the Materials and methods). Without RF-FG_STC_, the mean correlation across the effective neurons between the model responses and recorded spike rates was 0.24 with the noise-corrected explained variance [47] of 0.19 (Fig 9(B)). A relatively low correlation was expected since the modulation of the individual neurons was very weak [24]. The distribution of the difference in the correlation between the two models was centered around zero (Fig 9(C)), indicating the ineffectiveness of the RF-FG_STC_s in response to the figure and ground. This result suggests spatial variance of the FG structure, which seems meaningful for the segregation of figures from the ground.

## Discussion

To clarify the nature of FG-dependent responses in V4, we estimated the spatial structure of FG in natural image patches and their silhouettes that evoked the responses. Although Yamane *et al*. [24] reported FG-dependent responses, they focused on the FG at the CRF center, whether the figure or ground of an image was projected onto the CRF center. Therefore, the spatial structure of FG that evoked the neural responses has not yet been clarified. To elucidate the FG structure, we proposed combining the neural responses to natural and silhouette patches with the local FG structure based on spike triggered analyses. Weighted by the spike count observed in response to natural image stimuli, the corresponding FG labels were averaged to estimate the regions responsive to FG (RF-FG_STA_s). Approximately 50% of the examined neurons showed significant RF-FG_STA_s, and most of them exhibited antagonistic structures: a subregion responsive to a preferred side around the CRF center and a subregion responsive to a non-preferred side in the surroundings. The RF-FG_STA_s showed good agreement with those for the ideal FG responses, indicating that these neural responses were indeed dependent on the FG configuration projected on and around the CRF. The extents of figure-responsive subregions were smaller than those of ground-responsive subregions, indicating an agreement with the Gestalt law in figure perception. The results also suggested the preference for convexity in figure-responsive subregions.

The RF-FG_STA_s estimated from the filled patches would be straightforward since the presented stimuli and the FG labels were identical or contrast-reversed images. Notably, the RF-FG_STA_s estimated from the natural and filled-image patches shared the same structure. Since the natural image stimuli were distinct from the FG labels, this result supports that the neurons were, in fact, capable of determining FG from natural images. Although the natural image stimuli have very different textures and colors among each other, the estimated structures were similar to those estimated from the filled images, suggesting the invariance to textures and colors in the FG determination. The RF-FGs estimated by AF shared the same structure as the RF-FG_STA_s, supporting the veridicality of the STA. We estimated RF-FG based on STC and constructed computational models of individual neurons based on RF-FG_STA_ and RF-FG_STC_. The responses of the models based on both STA and STC did not show significant differences from those based solely on STA, suggesting the insubstantial contribution of nonlinearity and the spatial variance of the FG structure. These results indicate that the spatial organizations of figures and grounds in natural patches modulate the responses of V4 neurons.

In essence, the structures of the estimated kernels reflect the nature of the presented natural image patches but were limited by the presented stimuli since the number of stimuli was finite. We selected stimuli so that their degrees of convexity, closedness, and symmetry were widely distributed as evenly as possible. This selection is considered advantageous in spike triggered analyses, compared with not only to conventional artificial stimuli but also to ordinary natural movies. Because FG labels were not uniform, the mean FG label was subtracted from the estimated RF-FGs. The mean label was equivalent to an RF-FG in which the neural responses are equal across all stimulus images, and thus the subtraction represents the cancelation of bias evoked by the nonuniformity of FG organization. Another prominent characteristic of the stimuli was that the boundary between the figure and ground regions passed through the patch center. Although this constrain is crucial in assuring an appropriate distribution of figure and ground regions, it confines the structure of RF-FGs. If the neural responses were FG dependent and indicated a preference to figure, a subregion responsive to figure would appear in and around the CRF. This figure subregion may extend up to the patch center but not exceed the center because of this boundary constraint. To evaluate this RF-FG, a comparison with the ideal RF-FG is advantageous. If the neuron responded to any figure but not to the ground, the ideal RF-FG presents a clear antagonistic structure with respect to the patch center (Fig 4). In contrast, if the neuron responded to any stimuli without FG dependence, a uniformly zero RF-FG appears. A comparison with the ideal RF-FG provides the degree of FG dependence and the extent of subregions with respect to the ideal ones.

Although compensation for nonwhite stimuli has been proposed for estimating the RF structure of V1 neurons in response to natural movies, such compensation is applicable only to luminance. It could be theoretically possible to apply compensation to the direction and shape of the figure. However, the diminishment of autocorrelation results in the destruction of the FG structure. Intuitively, a figure region would be separated into many pieces and distributed randomly, or a contour around the figure would remain; thus, it no longer represents a *figure* in perception. The bias originated from non-zero correlation is inevitable in the present kernels; therefore, discussions on detailed structures of the kernels need to be cautious; however, our computational analysis indicated a good degree of validity in terms of whiteness by comparing the RF-FGs computed from the filled stimuli and the dot stimuli with whiteness (similarity = 0.87). A prominent difference between the patch and dot stimuli was the spatial resolution in RF-FGs. The computational analysis showed that an RF-FG whose extent was half of the mean extent of the CRF yielded a fair similarity (0.83) between the RF-FGs computed from the filled and dot stimuli. A visible difference may be found in the sharpness around peripheral boundary where the RF-FGs computed from the FG labels appear blur. Although the limited spatial resolution of FG labels appears to be an origin of the difference, other biases in the spatial structure of FG labels might also cause the difference. For instance, a set of FG labels with translations produced less blur in periphery (refer to S9 Fig). Because the frequency spectra of the original and translated labels were identical, other structural features seem to influence the estimation. Compared with the estimated extents of the individual CRFs and surrounding regions in V1 simple cells, which appeared rather noisy without intensive averaging [26, 43, 48], the inaccuracy originating from the nonwhiteness of our stimuli would not be substantial. Specifically, the structures including antagonism and relative extent, which were derived from the comparison with the ideal RF-FGs, were expected to be reliable. We also examined the veridicality of RF-FG_STA_ by comparison with RF-FG_AF_, which did not require whiteness. In contrast, a similar examination of the veridicality of RF-FG_STC_s has not been performed. It could be possible to compare the nonlinear RF-FGs derived from other methods, such as those using information theory [32]. However, it is not straightforward to compare two sets of multiple subunits that could represent the structure in different combinations. Therefore, we used RF-FG_STC_s in the context of estimating the degree of nonlinearity and did not take into account their spatial structures.

The estimated RF-FGs provide some clues about the neural mechanisms underlying FG determination. The mean STA across neurons (Fig 4) showed a slight bias of the figure region in the right and upper-right side. The bias of the CRF centers of recorded neurons relative to the fovea might explain this bias. The recorded cortical area corresponds to the lower visual field, and more than a half of the neurons were recorded from the right cortical hemisphere, meaning that the RF positions of neurons are biased to be lower left relative to the fovea. There is a possibility that more neurons code the figure in the upper right because the figures (objects) are placed on to the fovea in natural conditions. When we included neurons with significant FG-modulation as determined based on ANOVA with spike counts [24] but without effective RF-FG as determined based on the convergence and magnitude, the mean correlations between the estimated and ideal RF-FGs (Figs 2(D) and 3(D)) were increased. This result suggests that the present criteria for an effective RF-FG were somewhat stricter than expected. In contrast, some neurons that exhibited effective RF-FGs did not show significant FG-modulations. Although interpretation of these results is not straightforward, they might indicate that the neurons are responsive to a particular FG organization rather than a dichotomy of figure or ground at the CRF. The correlation between the responses of the STA-models and neurons was relatively low (0.24 across neurons). This result is consistent with the relatively low correct-rate of individual neurons in FG determination [24]. Our previous study reported that the integration of responses from a few tens of neurons achieved up to 85% correct. It is expected that the integration of multiple RF-FG models yields relatively high consistency with neural responses, which would support the distributed representation or population coding of figure and “proto object”. With STC analyses, we examined the contribution of nonlinearity. The nonlinearity assumed here is the invariance to spatial position and FG contrast, similar to a V1 complex cell responding to a grating pattern with different spatial phases and contrast polarities. However, these are not only the nonlinearities that we should consider. For example, the sudden increase in the predicted spike rate (Fig 8) is another signature of nonlinearity. In this case, the neurons respond only to a particular FG configuration but not to others. It may be possible to describe the response of each neuron by a linear-nonlinear (LNL) model that combines linearity and nonlinearity [49].

Understanding the interaction within or across subregions is crucial for modeling the neural mechanisms. The present RF-FGs were estimated by the presentation of local FG patterns that included single figure-regions except for few patches, and thus the RF-FGs can be considered as the first-order approximation of the responsive fields and cannot directly estimate the interaction. Physiological studies have reported a variety of interactions including surround suppression [43] and colinear facilitation [25]. FG-responsive neurons are also expected to exhibit some kind of interactions such as inter-figure suppression. A possible mechanism to realize the inter-figure suppression is based on the assumption of independent pathways for figure and ground. Intuitively, this mechanism allows the model to suppress the response to the FG pattern by a part of the F region in the stimulus. Though the model would be highly hypothetical without physiological and psychophysical evidence, yet suggestive for the investigation of suppression.

Further investigations to identify what contour shapes and textures tend to evoke the neural responses that signal figures and grounds are expected. For instance, contour shapes and high-contrast textures are good candidates to examine the relevance to neural responses. The integration and interaction of contour shapes and surface textures are also crucial in the examination [50]. It is also important to investigate the interactions with depth cues. The distinction of the contributions of the CRF and the surrounding surround modulation also provide useful information. Although the determination of CRF is challenging since the optimal stimuli for individual V4 neurons vary substantially, the distinction is expected to provide useful information on neural modeling. These studies would reveal the neural mechanisms underlying FG segregation. Moreover, the anatomical configuration of these neurons, such as the interaction among them and the clusters based on similarity in RF-FG, is certainly interesting. These questions are listed as important future works. The present study focused on local natural patches and attempted to exclude the influence of global and high-level information such as closedness, familiarity, and knowledge. The present data were recorded under weak anesthesia, which might have contributed to reducing feedback from higher-level cortical areas. A recent study reported that the curvature selectivity observed in monkey V4 was not affected by anesthesia [51]. Since curvature is a crucial local-cue for the determination of FG in filled stimuli, weak anesthesia might not alter local FG processing. Recordings with awake animals are expected to further clarify perceptual organization. Combining data under anesthesia and arousal would provide hints on top-down influences. Investigations of the neural responses to global natural scenes are also expected to clarify the neural mechanisms underlying perceptual organization. The combination of the responses to local patches and global scenes is expected to provide clues on the modulation of local information by global information. Further investigations would elucidate how the visual system gradually constructs the complex world in cortical areas [52].

## Supporting information

S1 Fig Stimulus set. The natural- and filled-image patches are shown next to each other. The mean filled-patches (FG-labels) are shown in the right-bottom. Positive and negative values (reddish and bluish colors) indicate the bias towards figure and ground regions, respectively. We observed a maximum bias of 16% in peripheral.
(TIF)

S2 Fig The model for the evaluation of stimulus set. The validity of the patch stimuli was computationally evaluated by comparing the RF-FG*s computed from the filled stimuli without whiteness and the dot stimuli with it. The model consisted of two stages: RF-FG and rectification. Illustrations of the model for the filled and dot stimuli are shown in (A) and (B), respectively. The RF-FGs in the models were given by the RF-FG_STA_s that were estimated from the neural data (Fig 2(A)). Note that the aim of the model was to examine the validity of the stimuli, whether the stimuli could be considered as pseudo-white, but not to propose neural mechanisms underlying FG processing. An input stimulus was multiplied pixelwise with the RF-FG_STA_, and then the sum of the products passed through a half-wave rectification. Stimuli were either the filled stimuli that were used in our experiments or randomly placed single dots (1×1 pixel). Both types of stimuli consisted of regions (dos) with +1 and -1 which corresponded to figure and ground, respectively. The filled stimuli included the mirror patches but not contrast-reversed patches for the sake of simplicity. The dot stimuli were unrealistic in natural scenes but satisfied whiteness; and thus, they were ideal for examining of the validity of the patch stimuli for STA. We computed the RF-FG*s from the filled and dot stimuli by the proposed STA method and compared the RF-FG*s to evaluate the validity of the patch stimuli.
(TIF)

S3 Fig Similarity between the simulated FG-RF*s computed from the filled and dot stimuli. We examined whether the predetermined RF-FG_STA_s in the models were correctly reproduced from the filled stimuli by the proposed STA method through the simulations of the model given in S2 Fig. The simulated RF-FG*s of three example model cells with distinct predetermined RF-FG_STA_s are shown in (A). The left column shows the predetermined RF-FG_STA_s. The yellow and blue colors indicate figure- and ground-preferring regions, respectively, with darker colors indicating greater magnitudes. The magnitudes were normalized by the maximum. The middle and the right columns show the RF-FG*s computed from the dot and patch stimuli, respectively. The mean cosine similarity across the examined cells between the predetermined RF-FG_STA_s and the simulated RF-FG*s computed from the dot stimuli was 0.99. This almost perfect similarity was expected since the set of the dot stimuli was an approximation of white noise. The right column shows the RF-FG*s computed from the filled stimuli, with the cosine similarity to that computed from the dot stimuli. We compared the RF-FG*s computed from the filled and dot stimuli. The distribution of the cosine similarities between the RF-FG*s is shown in (B). The median of the cosine similarities was 0.88 (mean = 0.87, SD = 0.062) across the examined cells, indicating a good validity of the patch stimuli. The models with predetermined RF-FGs whose profiles were given by the odd-symmetric difference-of-Gaussians yielded similar cosine similarities to those computed from the models with RF-FG_STA_s estimated from the neural data.
(TIF)

S4 Fig RLS algorithm for computing the adaptive filters. Adopted and modified from [35]. The final output is given by *w*(*N*+1). Free parameters were determined from experience: *δ*^-1^ = 0.00001, *μ* = 0, *β* = 0.99, and Q = 100.
(TIF)

S5 Fig RF-FG_STA_ from filled stimuli. Estimated RF-FG_STA_ computed from the filled stimuli for all neurons examined. The same conventions as Fig 2.
(TIF)

S6 Fig Comparison between the estimated and ideal RF-FGs for example neurons. The estimated and ideal RF-FGs for ground-preferred neurons for filled (A) and natural stimuli (B), and neurons with insignificant FG-modulation for filled (C) and natural stimuli (D). The same conventions are used as in Fig 2(C).
(TIF)

S7 Fig Estimated RF-FG_AF_s computed from the natural stimuli. The same conventions are used as in Fig 3(A) Estimated RF-FG_AF_s of the examined neurons. (B) RF-FG_STA_s (top) and RF-FG_AF_s (bottom) of three example neurons with the cosine similarity between the two. (C) Distribution of the cosine similarity between the RF-FG_STA_ and RF-FG_AF_. The RF-FG_STA_ and RF-FG_AF_ computed from the natural stimuli exhibit characteristics similar to each other, as observed with filled stimuli in Fig 5.
(TIF)

S8 Fig Illustration of RF-FG_STA_ and RF-FG_STC_s within a virtual two-dimensional space. Black and red dots represent stimuli, with a blue dot representing their mean. Red dots show a set of stimuli that evoked responses to a neuron. A blue arrow points to the mean of the responded stimuli (STA), and reddish arrows represent the variances across the responded stimuli in different orientations (STC).
(TIF)

S9 Fig FG-RF*s estimated from the filled stimuli with translations. The RF-FG*s computed from a set of filled patches including translations. The details of the stimulus configurations were described elsewhere [24]. The numbers in the right column show the cosine similarity between the RF-FG*s estimated from the filled stimuli with translations and the dot stimuli (refer to S3 Fig). The same conventions are used as in S3 Fig.
(TIF)
