# Supplementary figures and images for: Figure-ground responsive fields of monkey V4 neurons estimated from natural image patches
Source: PLoS One. 2022 Jun 16;17(6):e0268650. doi: 10.1371/journal.pone.0268650 (PMC9202882; doi:10.1371/journal.pone.0268650)

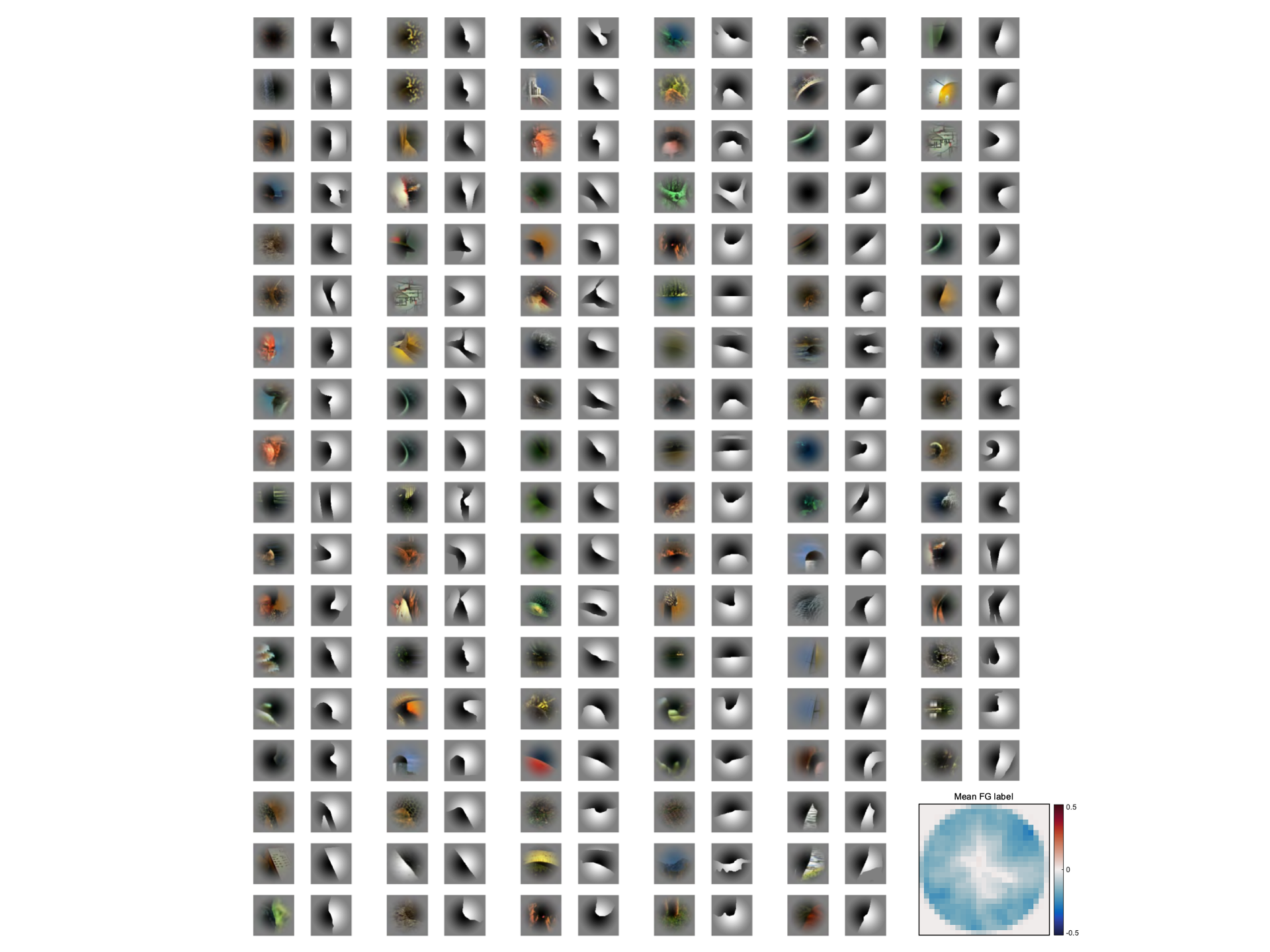

Supplement: S1 Fig — The natural- and filled-image patches are shown next to each other. The mean filled-patches (FG-labels) are shown in the right-bottom. Positive and negative values (reddish and bluish colors) indicate the bias towards figure and ground regions, respectively. We observed a maximum bias of 16% in peripheral. (TIF) [file pone.0268650.s001.tif]

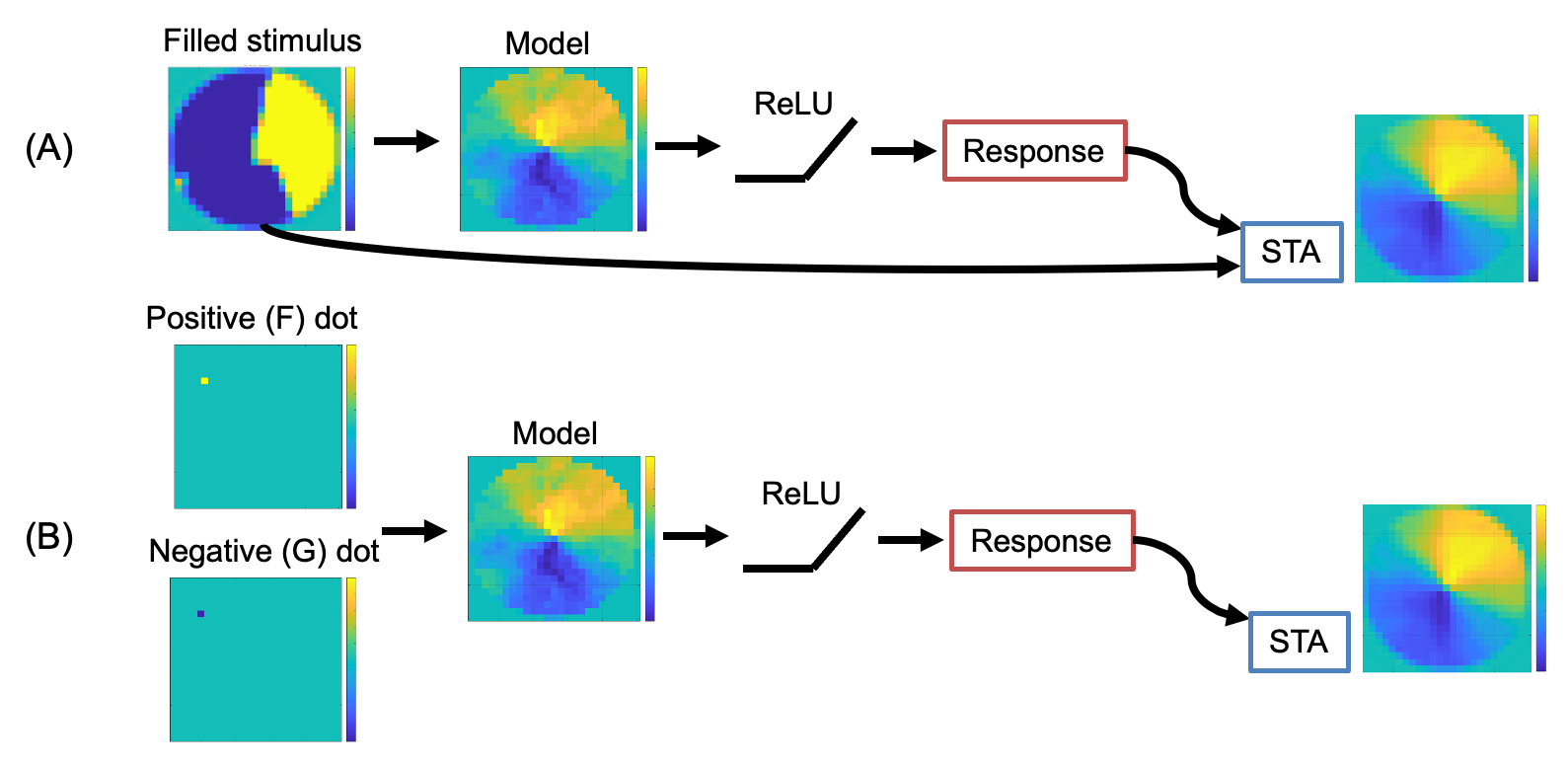

Supplement: S2 Fig — The validity of the patch stimuli was computationally evaluated by comparing the RF-FG*s computed from the filled stimuli without whiteness and the dot stimuli with it. The model consisted of two stages: RF-FG and rectification. Illustrations of the model for the filled and dot stimuli are shown in (A) and (B), respectively. The RF-FGs in the models were given by the RF-FGSTAs that were estimated from the neural data (Fig 2(A)). Note that the aim of the model was to examine the validity of the stimuli, whether the stimuli could be considered as pseudo-white, but not to propose neural mechanisms underlying FG processing. An input stimulus was multiplied pixelwise with the RF-FGSTA, and then the sum of the products passed through a half-wave rectification. Stimuli were either the filled stimuli that were used in our experiments or randomly placed single dots (1×1 pixel). Both types of stimuli consisted of regions (dos) with +1 and -1 which corresponded to figure and ground, respectively. The filled stimuli included the mirror patches but not contrast-reversed patches for the sake of simplicity. The dot stimuli were unrealistic in natural scenes but satisfied whiteness; and thus, they were ideal for examining of the validity of the patch stimuli for STA. We computed the RF-FG*s from the filled and dot stimuli by the proposed STA method and compared the RF-FG*s to evaluate the validity of the patch stimuli. (TIF) [file pone.0268650.s002.tif]

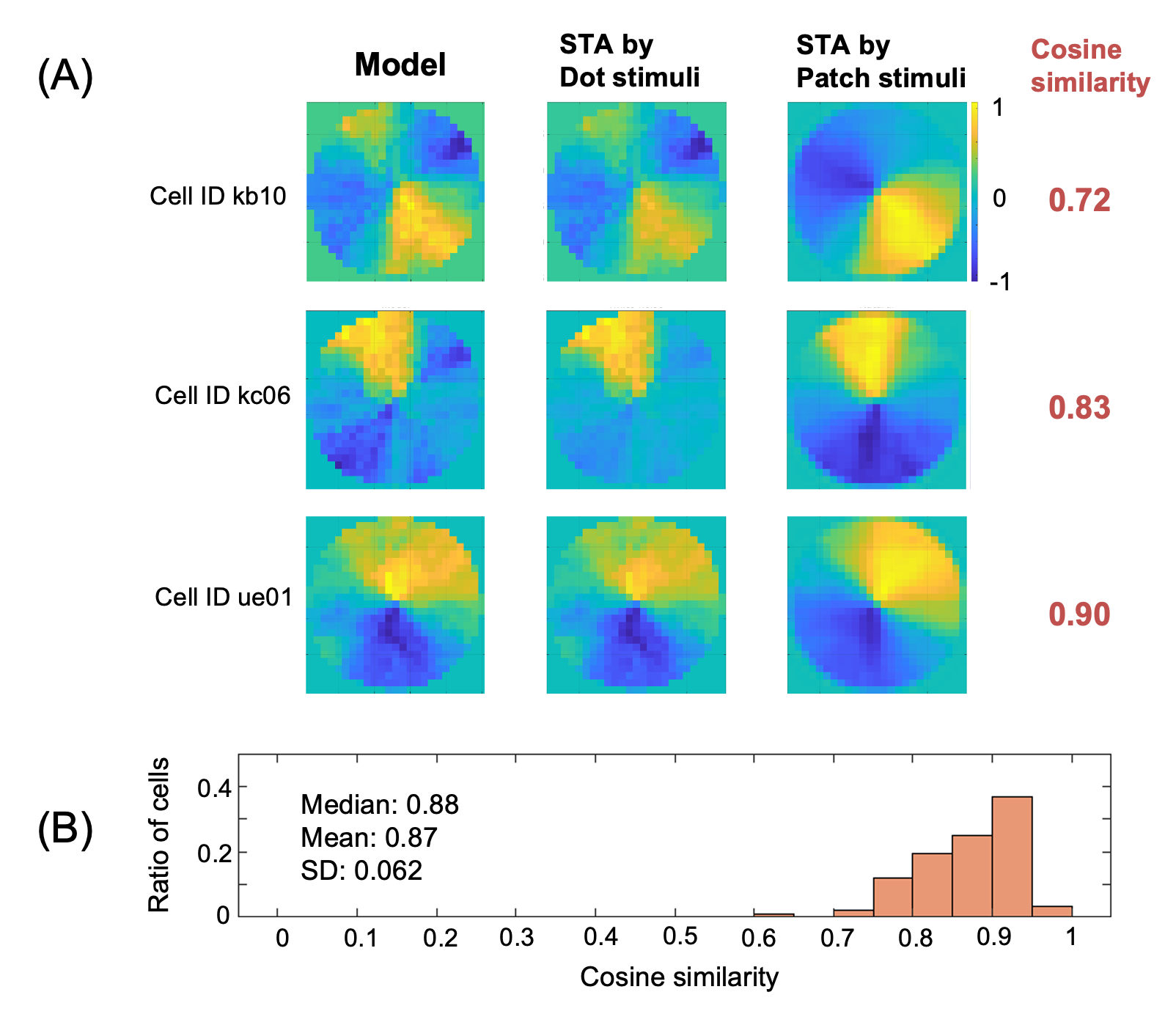

Supplement: S3 Fig — We examined whether the predetermined RF-FGSTAs in the models were correctly reproduced from the filled stimuli by the proposed STA method through the simulations of the model given in S2 Fig. The simulated RF-FG*s of three example model cells with distinct predetermined RF-FGSTAs are shown in (A). The left column shows the predetermined RF-FGSTAs. The yellow and blue colors indicate figure- and ground-preferring regions, respectively, with darker colors indicating greater magnitudes. The magnitudes were normalized by the maximum. The middle and the right columns show the RF-FG*s computed from the dot and patch stimuli, respectively. The mean cosine similarity across the examined cells between the predetermined RF-FGSTAs and the simulated RF-FG*s computed from the dot stimuli was 0.99. This almost perfect similarity was expected since the set of the dot stimuli was an approximation of white noise. The right column shows the RF-FG*s computed from the filled stimuli, with the cosine similarity to that computed from the dot stimuli. We compared the RF-FG*s computed from the filled and dot stimuli. The distribution of the cosine similarities between the RF-FG*s is shown in (B). The median of the cosine similarities was 0.88 (mean = 0.87, SD = 0.062) across the examined cells, indicating a good validity of the patch stimuli. The models with predetermined RF-FGs whose profiles were given by the odd-symmetric difference-of-Gaussians yielded similar cosine similarities to those computed from the models with RF-FGSTAs estimated from the neural data. (TIF) [file pone.0268650.s003.tif]

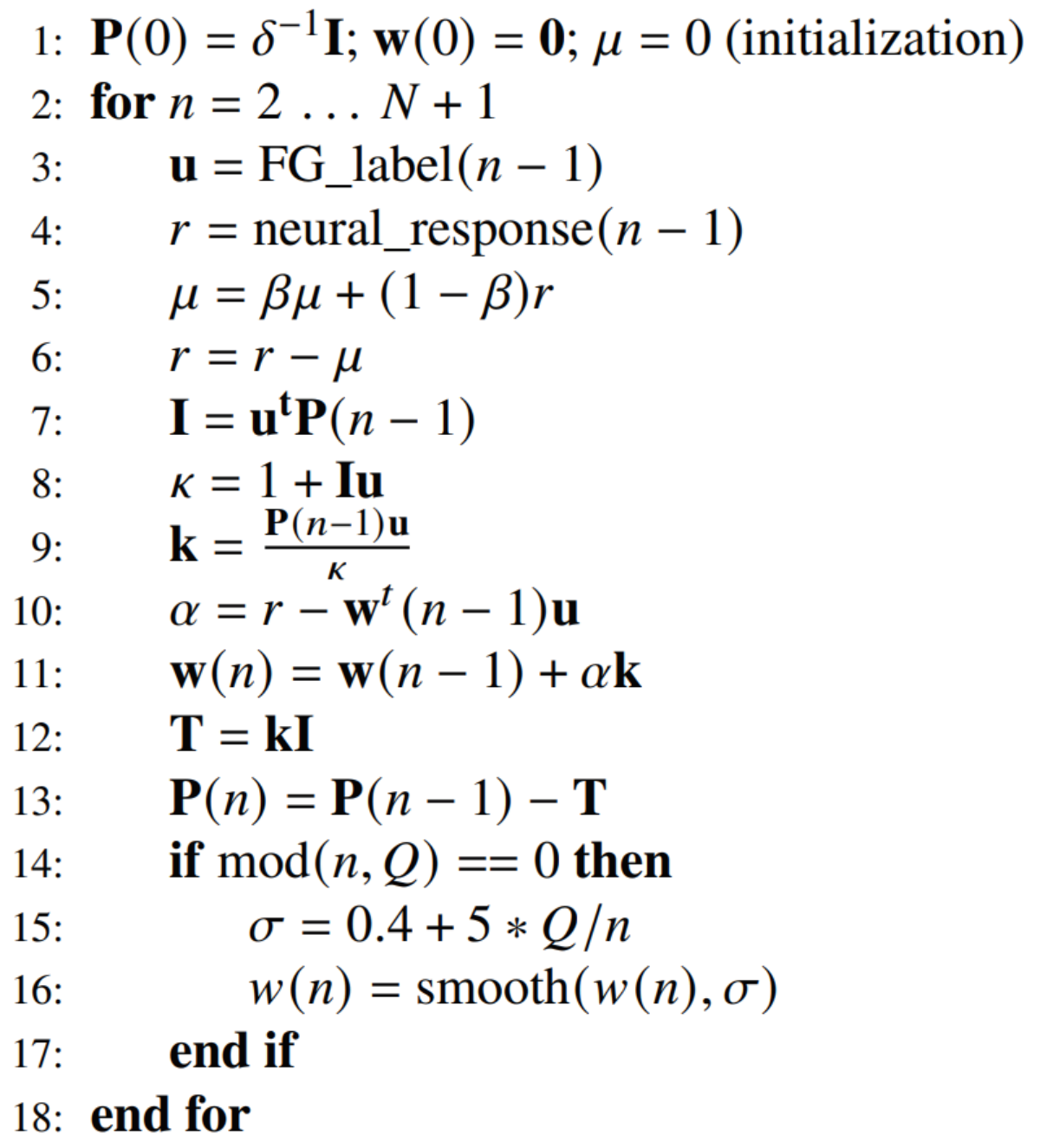

Supplement: S4 Fig — Adopted and modified from [35]. The final output is given by w(N+1). Free parameters were determined from experience: δ-1 = 0.00001, μ = 0, β = 0.99, and Q = 100. (TIF) [file pone.0268650.s004.tif]

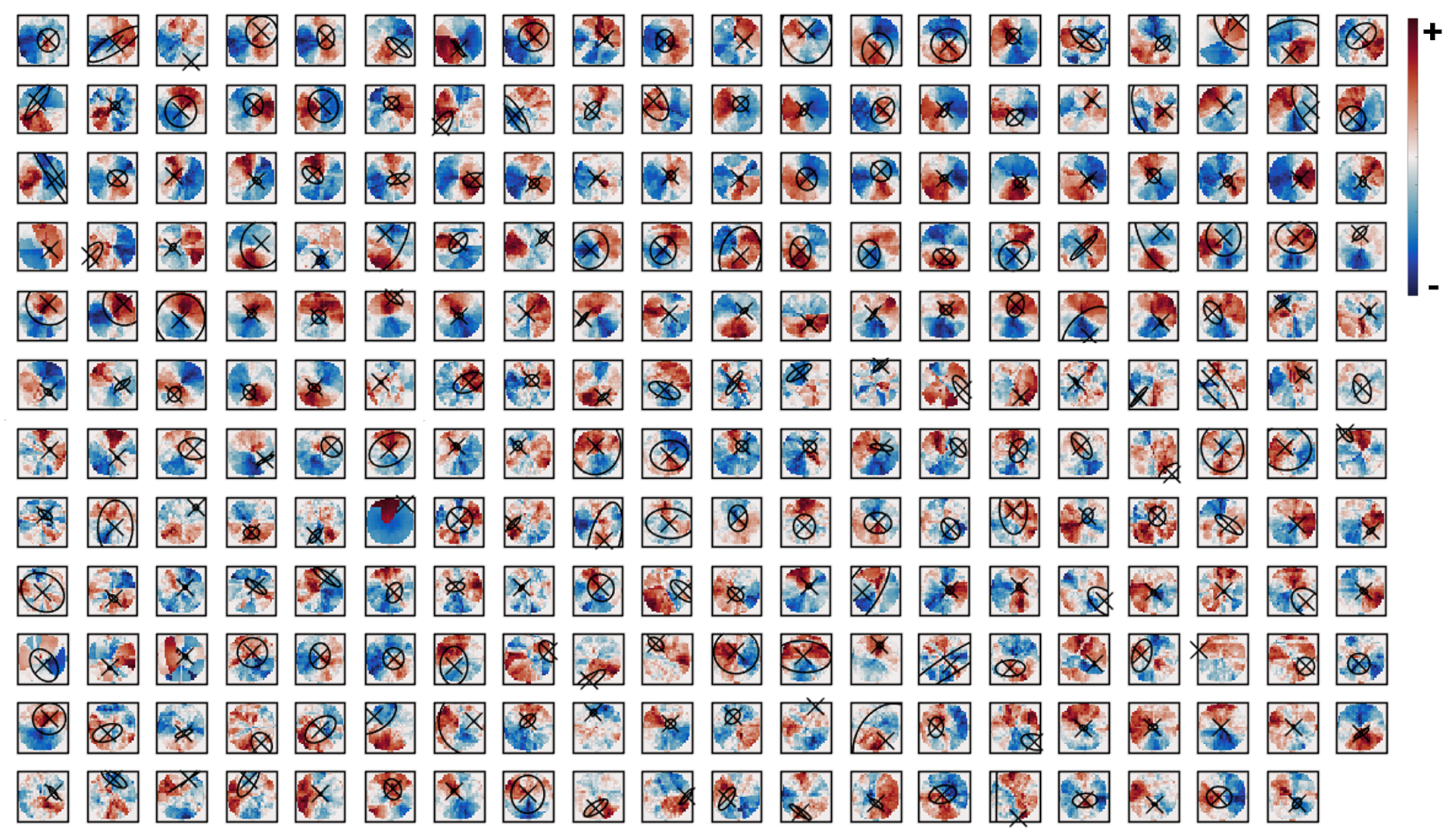

Supplement: S5 Fig — Estimated RF-FGSTA computed from the filled stimuli for all neurons examined. The same conventions as Fig 2. (TIF) [file pone.0268650.s005.tif]

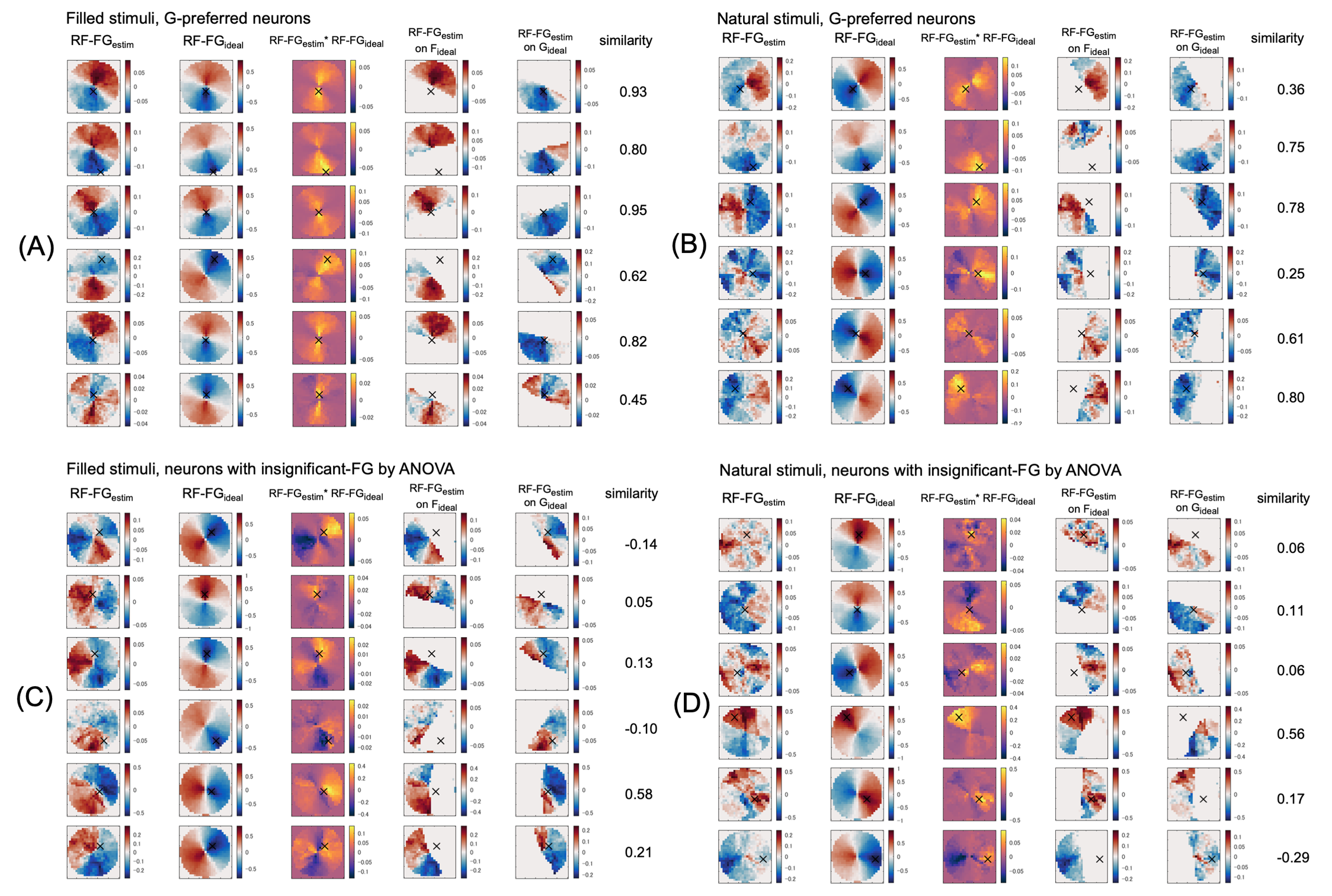

Supplement: S6 Fig — The estimated and ideal RF-FGs for ground-preferred neurons for filled (A) and natural stimuli (B), and neurons with insignificant FG-modulation for filled (C) and natural stimuli (D). The same conventions are used as in Fig 2(C). (TIF) [file pone.0268650.s006.tif]

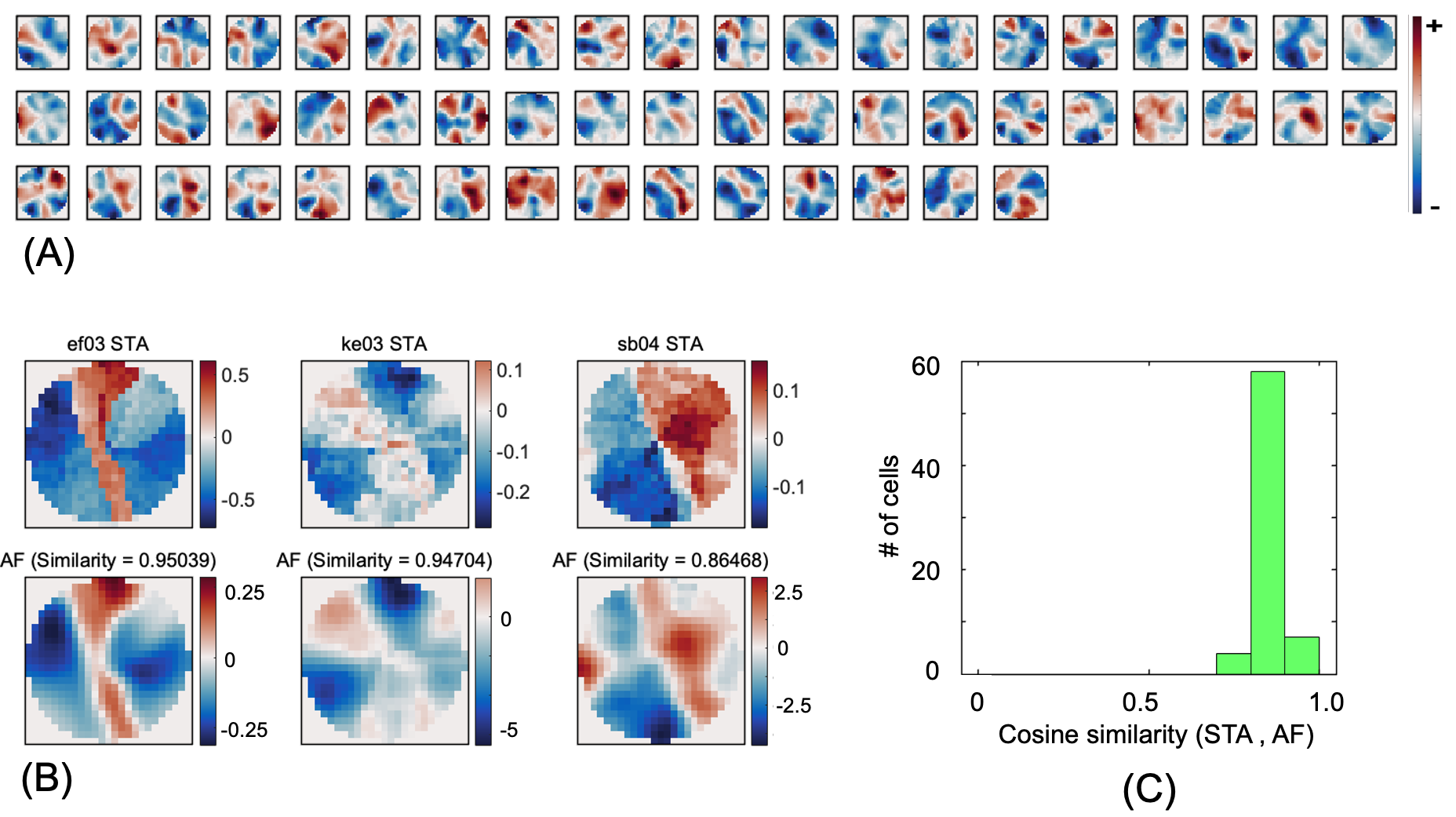

Supplement: S7 Fig — The same conventions are used as in Fig 3(A) Estimated RF-FGAFs of the examined neurons. (B) RF-FGSTAs (top) and RF-FGAFs (bottom) of three example neurons with the cosine similarity between the two. (C) Distribution of the cosine similarity between the RF-FGSTA and RF-FGAF. The RF-FGSTA and RF-FGAF computed from the natural stimuli exhibit characteristics similar to each other, as observed with filled stimuli in Fig 5. (TIF) [file pone.0268650.s007.tif]

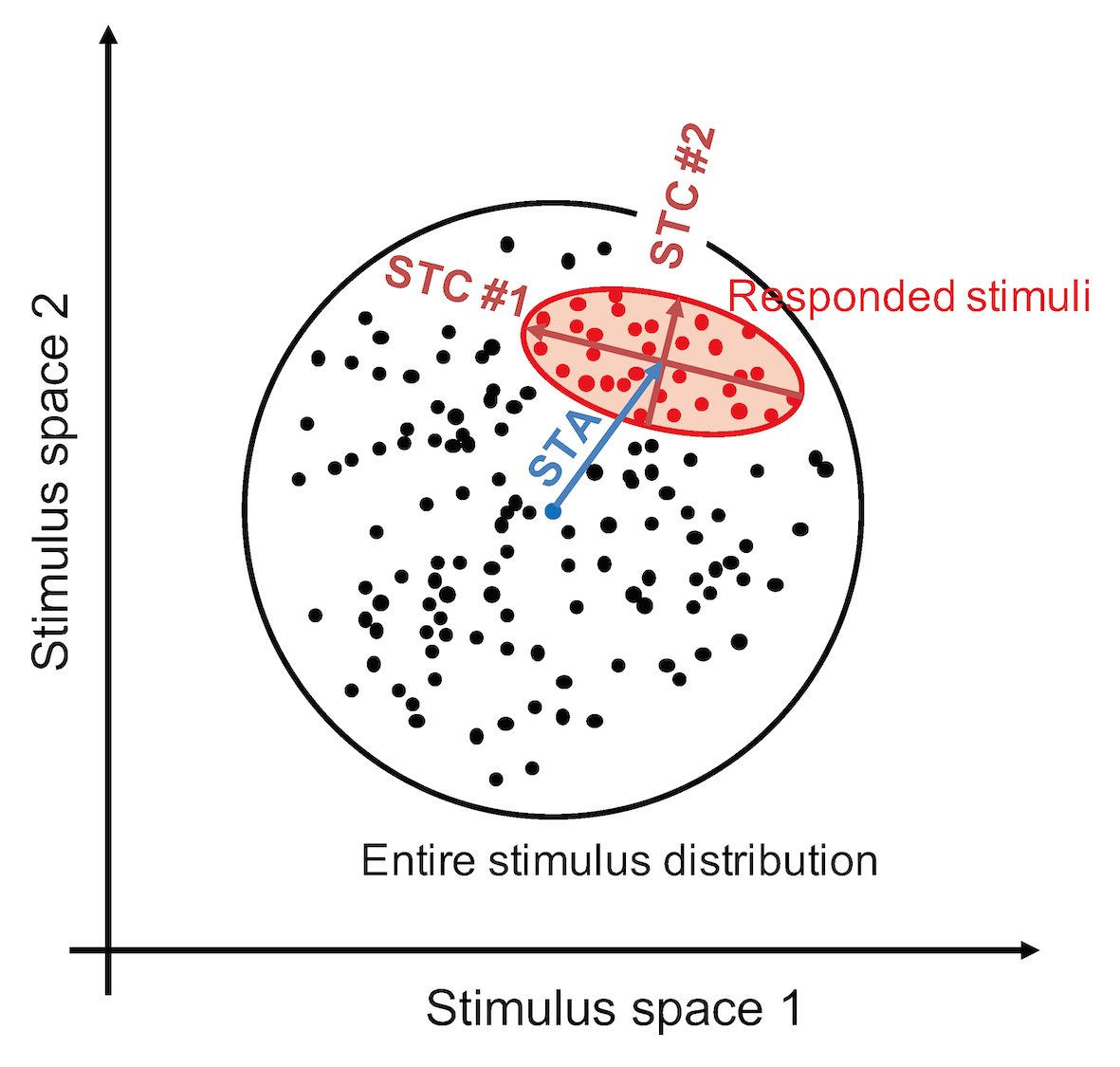

Supplement: S8 Fig — Black and red dots represent stimuli, with a blue dot representing their mean. Red dots show a set of stimuli that evoked responses to a neuron. A blue arrow points to the mean of the responded stimuli (STA), and reddish arrows represent the variances across the responded stimuli in different orientations (STC). (TIF) [file pone.0268650.s008.tif]

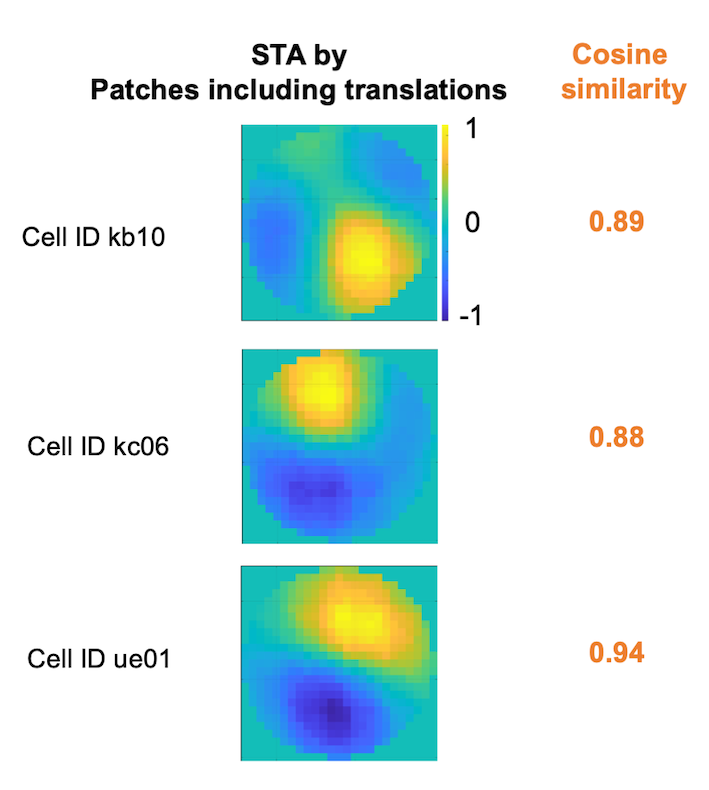

Supplement: S9 Fig — The RF-FG*s computed from a set of filled patches including translations. The details of the stimulus configurations were described elsewhere [24]. The numbers in the right column show the cosine similarity between the RF-FG*s estimated from the filled stimuli with translations and the dot stimuli (refer to S3 Fig). The same conventions are used as in S3 Fig. (TIF) [file pone.0268650.s009.tif]
